# Red blood cell phenotype fidelity following glycerol cryopreservation optimized for research purposes

**DOI:** 10.1371/journal.pone.0209201

**Published:** 2018-12-21

**Authors:** Stephen C. Rogers, Laura B. Dosier, Timothy J. McMahon, Hongmei Zhu, David Timm, Hengtao Zhang, Joseph Herbert, Jacqueline Atallah, Gregory M. Palmer, Asa Cook, Melanie Ernst, Jaya Prakash, Mark Terng, Parhom Towfighi, Reid Doctor, Ahmed Said, Matthew S. Joens, James A. J. Fitzpatrick, Gabi Hanna, Xue Lin, Julie A. Reisz, Travis Nemkov, Angelo D’Alessandro, Allan Doctor

**Affiliations:** 1 Department of Pediatrics, Divisions of Critical Care Medicine, Washington University in Saint Louis, School of Medicine, Saint Louis, MO, United States of America; 2 Department of Biochemistry & Molecular Biophysics, Washington University in Saint Louis, School of Medicine, Saint Louis, MO, United States of America; 3 Department of Pediatrics, Duke University School of Medicine, Durham, NC, United States of America; 4 Department Medicine, Duke University School of Medicine, Durham, NC, United States of America; 5 Departments of Medicine, Durham VA Medical Center, Durham, NC, United States of America; 6 Department of Radiation Oncology, Duke Univ. School of Medicine, Durham, NC, United States of America; 7 Washington University Center for Cellular Imaging, Washington University in Saint Louis, School of Medicine, Saint Louis, MO, United States of America; 8 Departments of Neuroscience and Cell Biology & Physiology, Washington University in Saint Louis, School of Medicine, Saint Louis, MO, United States of America; 9 Department of Biochemistry, University of Colorado Denver—Aurora, CO, United States of America; The Ohio State University, UNITED STATES

## Abstract

Intact red blood cells (RBCs) are required for phenotypic analyses. In order to allow separation (time and location) between subject encounter and sample analysis, we developed a research-specific RBC cryopreservation protocol and assessed its impact on data fidelity for key biochemical and physiological assays. RBCs drawn from healthy volunteers were aliquotted for immediate analysis or following glycerol-based cryopreservation, thawing, and deglycerolization. RBC phenotype was assessed by (1) scanning electron microscopy (SEM) imaging and standard morphometric RBC indices, (2) osmotic fragility, (3) deformability, (4) endothelial adhesion, (5) oxygen (O_2_) affinity, (6) ability to regulate hypoxic vasodilation, (7) nitric oxide (NO) content, (8) metabolomic phenotyping (at steady state, tracing with [1,2,3-^13^C_3_]glucose ± oxidative challenge with superoxide thermal source; SOTS-1), as well as *in vivo* quantification (following human to mouse RBC xenotransfusion) of (9) blood oxygenation content mapping and flow dynamics (velocity and adhesion). Our revised glycerolization protocol (40% v/v final) resulted in >98.5% RBC recovery following freezing (-80°C) and thawing (37°C), with no difference compared to the standard reported method (40% w/v final). Full deglycerolization (>99.9% glycerol removal) of 40% v/v final samples resulted in total cumulative lysis of _~_8%, compared to _~_12–15% with the standard method. The post cryopreservation/deglycerolization RBC phenotype was indistinguishable from that for fresh RBCs with regard to physical RBC parameters (morphology, volume, and density), osmotic fragility, deformability, endothelial adhesivity, O_2_ affinity, vasoregulation, metabolomics, and flow dynamics. These results indicate that RBC cryopreservation/deglycerolization in 40% v/v glycerol final does not significantly impact RBC phenotype (compared to fresh cells).

## Introduction

Red blood cell (RBC) cryopreservation was described almost seventy years ago[1] and subsequently explored as means to prolong RBC storage prior to transfusion[2, 3]. Of note, RBC cryopreservation has never been optimized to satisfy research requirements, which differ from those for clinical use.

The main challenge in cryopreservation (i.e., the use of very low temperature to preserve intact cells[4]) is not endurance of extremely low temperature, but rather that of an intermediate temperature zone (approx. between -10 to -60°C) to which cells are exposed twice–during cooling and warming[5]. This intermediate temperature zone may damage cells due to imbalanced transmembrane water transport, a temperature-dependent process[6]. If water is not given enough time to exit, injury occurs from intracellular ice formation (IIF), leading to mechanical shearing[6, 7]. However, if water exits too quickly, injury may result from cell shrinkage and related changes in intracellular solute concentrations (“solution effects” injury)[8, 9]. Between these two ends of the spectrum, optimal cooling and warming rates exist that minimize freezing injury[10, 11].

Cryoprotective agents (CPAs) modulate membrane water transport[4] and inhibit intracellular ice formation. The most widely used CPA for RBC cryopreservation is glycerol, which enters cells via facilitated transport[12], whilst remaining nontoxic at high concentrations[13]. Two RBC glycerol cryopreservation protocols have been developed[14]: the high-glycerol (HGM; ~40%-50% w/v)[15, 16] and low-glycerol methods (LGM; ~15–20% w/v)[17, 18] (Table 1). We focused on a HGM (40% v/v), due to material and equipment availability and simpler sample transport, on dry ice rather than in liquid nitrogen, which LGM requires.

**Table 1:** Comparison of the high-glycerol (HGM) and low-glycerol (LGM) methods for the cryopreservation of human RBCs.

CONDITIONS	HGM (~40–50% w/v)	LGM (~15–20% w/v)
Freezing Temperature	^-^80°C	^-^197°C
Freezing Rate	Slow (1C/min)	Fast
Freezing Technique	Mechanical freezer	Liquid nitrogen
Storage Temperature	Min. ^-^65°C	Min. ^-^120°C
Impact of temperature changes	Thawing/refreezing possible	Highly destructive
Transportation	Dry ice	Nitrogen vapor
Time to deglycerolize	Lengthy (60 min)	Moderate (30 min)
Typical lysis following thaw	1–5% [19–22]	2–20% [18, 23, 24]
Typical lysis following CPA removal	10–20% [19–23, 25–27]	5–13% [18, 23]

Adoption of glycerol-based RBC cryopreservation has been limited due to cost (money and time) of the deglycerolization process[28–30]. To prevent hypo-osmotic hemolysis during CPA removal, manual washing techniques have been developed using aqueous sugar[31] or salt[28]. Additionally, an automated system has been developed for clinical use[14], in which: (1) residual glycerol concentration is below threshold for hemolysis (of endogenous RBCs)[29], (2) final wash hemolysis is <1%, and (3) 24h post-transfusion RBC recovery is >75%, meeting current regulatory requirements[32]; however, total RBC loss is typically _~_15%[20]. This may be of clinical benefit, by culling damaged/senescent cells prior to transfusion. However, for research the goal is to obtain a RBC population identical to that circulating in subjects. Moreover, the most vulnerable cells are likely of greatest interest.

RBC assessment following cryopreservation has focused on basic *in vitro* analyses (hemolysis[2, 3, 21], morphologic[2, 33], rheologic[34], osmotic[2, 33, 34], surface marker[35], and biochemical[2, 3, 33]) and on *in vivo* survival[3, 21, 36], rather than on physiologic function (O_2_ transport, NO processing, vasoreactivity, and metabolism). Given new understanding of the pivotal role played by RBCs in vasoregulation[37], such analyses are relevant as well. Since such phenotypic analyses require intact cells, research has been limited by subject availability/proximity. To enable separation between subject and sample analysis, we identified need for research-optimized RBC cryopreservation, with simplified processing and maximized fidelity of recovered cells to *in vivo* phenotype. To achieve these goals, we developed a novel glycerolization procedure, minimizing front-end processing time. Additionally, we optimized a deglycerolization protocol, assessing sample osmolality and CPA removal at each step, thereby minimizing RBC lysis. Finally, to assess cryopreservation impact, we compared the phenotypic profile of cryopreserved/deglycerolized RBCs to that of fresh RBCs.

## Methods

### Blood sampling and preparation of RBCs

This project was approved by the Washington University and Duke IRBs and written informed consent was obtained from all participants. Venous samples (EDTA or heparinized) were drawn from healthy volunteers. Unless otherwise stated, whole blood was centrifuged (1,500–2,000*g*, 10min, 4°C); plasma supernatant and buffy coat were removed prior to RBC preparation. All subsequent steps were carried out at RT, given that glycerol flux into RBCs is temperature dependent[38].

### Standard glycerolization

**Fig 1:**
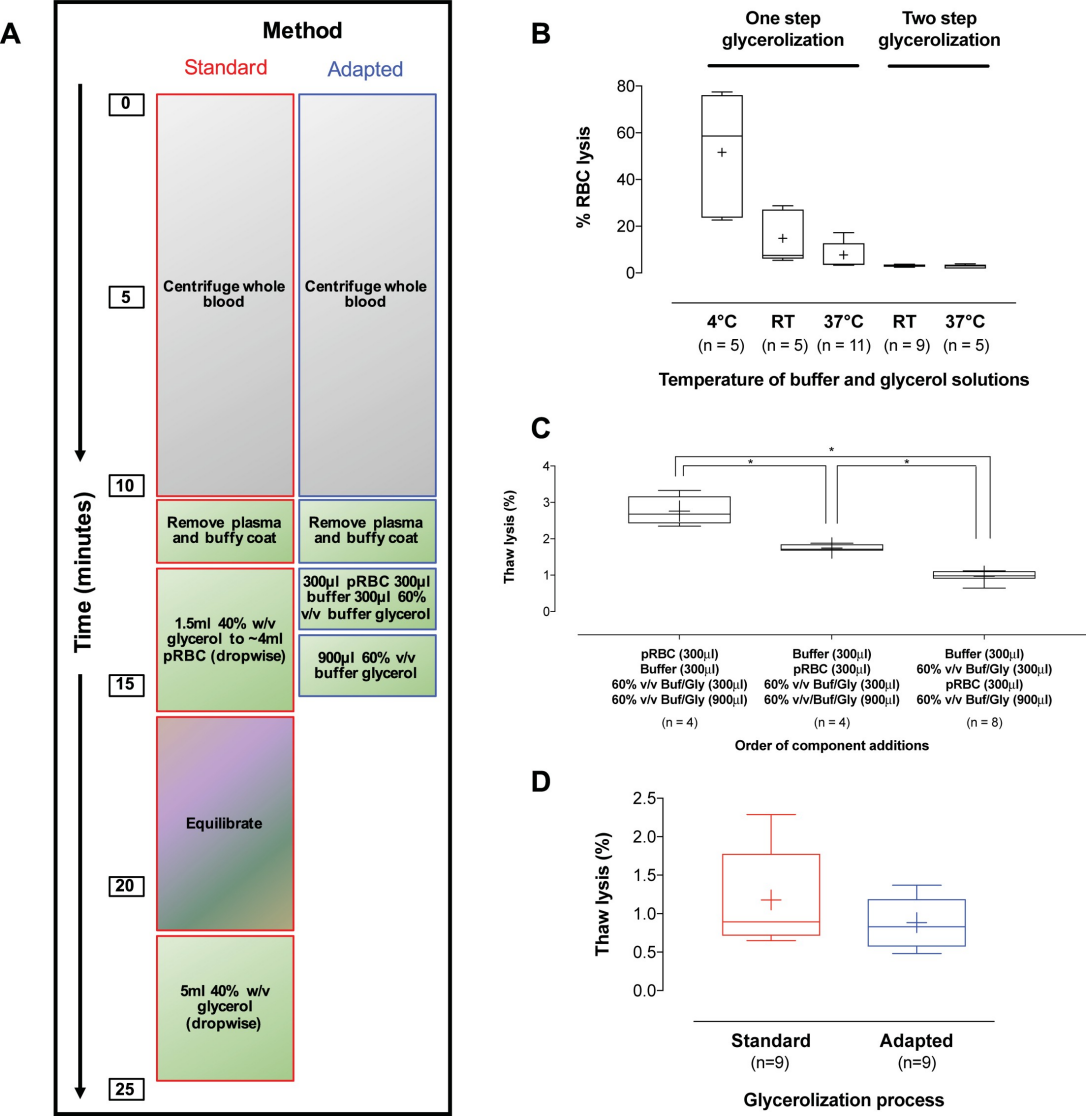
Optimization of an adapted glycerolization procedure and comparison to the standard reported literature method. **(A)** Diagram of the standard glycerolization protocol and our adapted method, designed to minimize sample processing time and allow collection/processing/storage of smaller RBC aliquots. In the standard glycerolization method, RBC volumes were added dropwise resulting in a final solution that was 40% w/v glycerol; in the adapted method, RBCs were added as a bolus, resulting in a final 40% v/v glycerol solution. All centrifugation was performed at 1,500*g*, 5min, at 4°C. **(B)** Whisker-plot comparing RBC lysis resulting from the addition of buffer/glycerol (60% v/v glycerol buffer stock added to samples) at different temperatures to pRBCs in a one step (bolus addition; 300μl pRBC to 600μl buffer/glycerol stock) or two step (300μl pRBCs to 300μl buffer to 300μl buffer/glycerol stock = step one, followed by addition of further 900μl buffer/glycerol stock = step two) process. Note, both procedures resulted in a 40% v/v glycerol final. Additionally in this experiment, no samples were frozen. Glycerol solutions were added and then samples immediately centrifuged and lysis measured. **(C)** Whisker-plot comparing the order of addition of pRBC, buffer, and buffer/glycerol (60% v/v glycerol buffer stock added to samples) in the two step glycerolization procedure, on the amount of lysis following sample freezing at -80°C and thawing for 2 minutes at 37°C. Order of addition of components is highly important. **(D)** Whisker-plot comparing RBC lysis following sample freezing and thawing, for the standard (40% w/v glycerol final) and our adapted (40% v/v glycerol final) glycerolization procedure. Mean RBC lysis was not different between the two methods; however, our adapted glycerolization procedure was 40% more time efficient (n = 9 matched samples from 3 individual blood donors).

We followed a scaled-down step-wise cryopreservation procedure (40% w/v glycerol final), as reported[28, 38, 39] (S1 Extended Methods). Total processing time was ~25min (Fig 1A). Glycerolized samples were then placed into a Coolcell freezing container (Biocision, Larkspur, CA), ensuring rate-controlled freezing (-1°C/min when placed into a -80°C freezer).

### Adapted glycerolization

We then modified the cryopreservation process to shorten processing and to allow collection of smaller sample volumes, more appropriate for research analysis (Fig 1A). Initial collection and centrifugation were unchanged from the standard protocol. Briefly, RBCs (300μl) were aliquoted from the bottom of the RBC pellet into cryopreservation tubes containing 300μl buffer and 300μl buffer/glycerol stock (60% v/v glycerol in starting buffer solution). Immediately thereafter, samples were mixed gently by repeated inversion, after which additional PBS-glycerol (900μl, 60% v/v glycerol in starting buffer solution) was added and the samples were again inverted (glycerol final 40% v/v; see S1 Extended Methods). Samples were then placed into a Coolcell freezing container. Matched fresh RBC samples were processed (washed 3X in PBS) for immediate analysis.

### Deglycerolization

Cryopreserved samples (40% v/v glycerol final) were thawed (37°C, 2min), inverted to mix, then transferred to Eppendorf tubes for centrifugation (1,500*g*, 5min, RT). The majority of supernatant (_~_1.4ml) was removed, leaving _~_100μl layering the RBC pellet (_~_300μl); this residual was inverted to gently resuspend RBCs (~ 75% Hct) for subsequent deglycerolization.

### Standard deglycerolization

**Fig 2:**
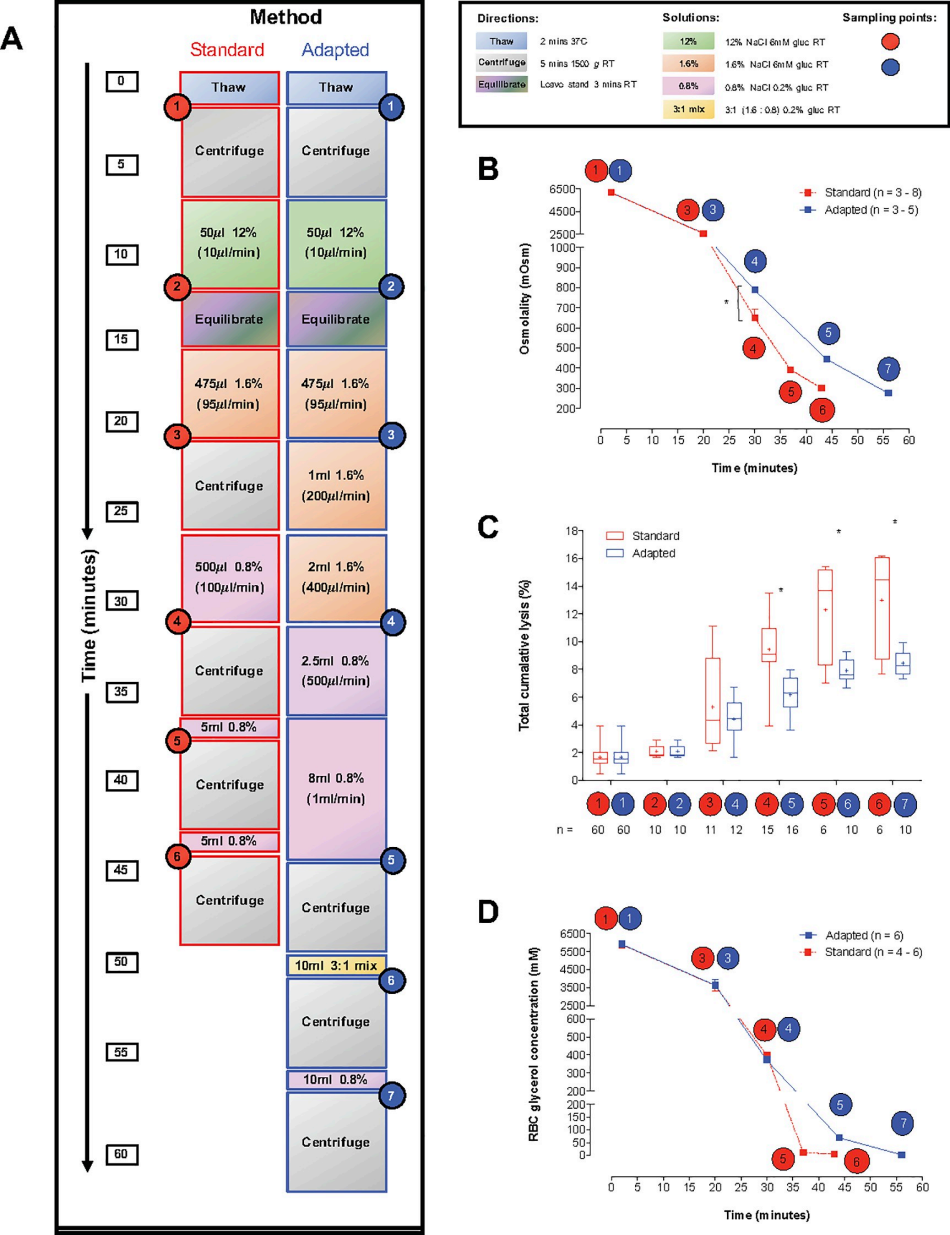
Comparison of the standard and our adapted deglycerolization procedure. **(A)** Diagram of the standard deglycerolization protocol and our adapted method, designed to reduce RBC lysis. Samples (40% v/v glycerol final) were thawed (2min, 37°C), then centrifuged (1,500*g*, 5min, 4°C). For equilibration, samples were left undisturbed for 3min at RT. Circled numbers identify sampling points for the graphs presented in B-D. **(B)** Sample osmolality was measured at various stages of the standard and adapted deglycerolization methods. The adapted method slowed osmolality reduction during de-gycerolization, thereby protecting cells from lysis (n = 3–8 samples from 3 individual blood donors). **(C)** Total cumulative RBC lysis measured at different stages of the standard and adapted deglycerolization methods. RBC lysis was significantly reduced using the adapted deglycerolization method (n = samples, shown on graph. Minimum number of individual blood donors = 4). **(D)** Intracellular RBC glycerol measured at different stages of the standard and adapted deglycerolization methods. Both methods result in total glycerol removal (> 99.9%) (n = 4–6 samples from 3 individual blood donors).

We consolidated the published standard serial dilution procedure[28, 39]; deglycerolizing solutions were: 12% NaCI, 1.6% NaCI (unbuffered), followed by isotonic wash (0.8% NaCI, 0.2% glucose (11 mM), pH 7.4) (Table 2). The process required ~50min (Fig 2A) (see S1 Extended Methods).

**Table 2:** Characteristics of the buffers used in sample glycerolization and deglycerolization.

Buffer	Molarity	NaCl (%)	Osmolality (mOsm)	pH
PBS-glycerol (60% v/v glycerol in PBS)	10 mM	-	8100	7.4
PBS	10 mM	-	280	7.4
Wash buffer 1 (12% NaCl)	2.05 M	12	3700	Unbuff
Wash buffer 2 (1.6% NaCl)	274 mM	1.6	530	Unbuff
Wash buffer 3 (0.8% NaCl + 0.2% glucose)	137 mM	0.8	290	7.4

### Adapted deglycerolization

We optimized the above method to minimize RBC lysis by extending the dilution sequence (reducing osmolality less rapidly). The process required ~1hr (Fig 2A) (see S1 Extended Methods).

### RBC lysis

To determine the extent of RBC lysis, we assayed Hb concentration ([Hb]) spectrophotometrically (Biotek μQuant, Winooski, VT or FLUOstar Omega, BMG Labtech, Cary, NC) in both the post-centrifugation supernatant (numerator) and the original RBC-glycerol suspension (denominator), accounting volumes of each, and multiplying by 100 to index hemolysis to the starting total [Hb]. [Hb] assays and calculation of lysis percentage were repeated at each stage following cryopreservation, at various points during deglycerolization (Fig 2A) and successive washes. An alternative lysis calculation is outlined in S1 Extended Methods. [Hb] values from supernatants and RBC lysates did not differ whether measured by simple spectrophotometric or Drabkin’s assays, using the following formula:

$$Heme\;(mM)=\frac{\left(sample\;absorbance\;@\;540\;nM)\;X\;(dilution\;factor\right)}{\left(extinction\;coefficient\;@\;540\;nM)\;X\;(pathlength\;correction\right)}$$

where the extinction coefficient was 11mM^-1^cm^-1^ (this extinction coefficient is for the product of the reaction between Drabkins reagent and hemoglobin, i.e., cyanomethemoglobin) and the pathlength correction was 0.8 for a 300μl volume in a 96-well plate.

### Sample osmolality

Osmolality was determined by freezing point depression (Osmette A, Precision Systems Inc, Natick, MA).

### Intracellular RBC glycerol concentration

Intracellular glycerol was determined using a Glycerol Assay kit (Sigma MAK117) and measured spectrophotometrically (A_570_; Biotek μQuant, Winooski, VT) (see S1 Extended Methods).

### Scanning electron microscopy

See S1 Extended Methods for details; briefly, RBCs were fixed to poly-L-lysine coated coverslips, dehydrated in ethanol, dried (Leica EM CPD 300, Vienna, Austria), mounted on aluminum stubs and sputter coated with iridium (Leica ACE 600, Vienna, Austria), then loaded into a FE-SEM (Zeiss Merlin, Oberkochen, Germany) and imaged (3KeV, 300pA) using the Everhart Thornley secondary electron detector. Cell morphology was quantitated using a standard method[40]. Briefly, three separate images of cells (~50–200 cells per image), either fresh, glycerolized, thawed, or deglycerolized, were counted and categorized according to shape, into one of six classes; (a) smooth disc (1.0), (b) crenated disc (0.8), (c) crenated discoid (0.6), (d) crenated spheroid (0.4), (e) crenated sphere (0.2), (f) smooth sphere (0). The percentage of each cell type was multiplied by the factor assigned to each cell type (in parentheses above), and the sum of these products was added together to calculate the morphological index (Im).

### Standard RBC indices (CBC)

Standard RBC parameters including: mean corpuscular volume (MCV), mean corpuscular hemoglobin (MCH), mean corpuscular hemoglobin concentration (MCHC), and red cell distribution width (RDW) were measured using a conventional analyzer (Coulter ACT diff2, Beckman, Brea, CA).

### Osmotic fragility

Washed RBCs (50μL) were suspended in NaCl solutions (5ml) ranging from 0-10g/L and incubated (30min, RT). Intact RBCs were pelleted (2,000*g*, 10min, 4°C) and supernatant absorbance was read (540nm, Biotek μQuant, Winooski, VT). 100% lysis was assigned to the 0g/L NaCl solution, against which others were indexed.

### RBC deformability

RBCs in PBS (20*μ*l, 40% Hct) were added to iso-osmolar polyvinylpyrrolidone (5ml, viscosity ~30mPa*s) (Mechatronics, Netherlands); RBC deformability (elongation index at 37°C and 0.3–30 Pa) was measured by ektacytometry (Laser-assisted Optical Rotational Red Cell Analyzer (LORRCA), RR Mechatronics, Hoorn, Netherlands).

### RBC adhesion (*ex vivo*)

RBC adhesivity to endothelium, in a custom variable height plexiglass flow chamber, was determined as described[41–44] (see S1 Extended Methods). Labeled RBCs (fluorescent dye PKH26) were introduced to the chamber (1.5ml/min), then allowed to dwell for 5–10min. The cell number at each location (height) was recorded. Then, 5–10min of fluidic flow was conducted with PBS at rates calculated to produce shear stress ranging 1–10dynes/cm^2^ and the number of adherent cells at each location was re-counted. Shear stress and percent adhesion were calculated at each height.

### O_2_ affinity

O_2_-hemoglobin dissociation curves (ODC) were determined *in vitro* (Hemox-Analyzer, TSC Scientific, New Hope, PA). The p50 (pO_2_ at which HbSO_2_ is 50%) was extrapolated; the Hill co-efficient was also calculated from these data (TCS Hemox Data Aquisition System, TCS Scientific Corp, New Hope, PA).

### RBC vasoactivity (hypoxic vasodilation, HVD)

Male New Zealand white rabbits (n = 7; 1.8-2kg; strain code 052, Charles River) were euthanized (IV pentobarbital). The aorta was harvested and endothelium-intact rings were prepared for isometric tension recordings (Radnoti vascular ring array, Harvard Apparatus, Holliston, MA): 2gm resting tension, 37°C, Krebs, as described[45]. For each experiment, eight rings were run in parallel; n = 1 represents data averaged from rings with functional endothelium (>60% ACh-induced relaxation following PE-induced pre-constriction)[45, 46]; percentage relaxation was calculated as the RBC-induced decrease in tension indexed to baseline plateau tension (during hypoxia; (95% N_2_ and 5% CO_2_)[46].

### RBC NO content

Hb from fresh, frozen (without cryopreservation) and cryopreserved RBCs (deglycerolized or not) was assayed for total, Fe-bound and thiol-bound NO (SNO) content by photolysis/chemiluminescence, as described[47]. No sample treatment was used, other than desalting (G25) and paired analyses with/without HgCl_2_ (6-fold molar excess over [Hb thiol])[48, 49].

### Metabolomics

Metabolomics analyses and tracing experiments with [1,2,3-^13^C_3_]glucose were performed, as described[50, 51]. Either washed fresh, or glycerolized, frozen, thawed and deglycerolized RBCs (denoted deglycerolized; DG) were incubated with [1,2,3-^13^C_3_]glucose in the presence of (i) PBS (control), (ii) DMSO (vehicle control), or (iii) SOTS1 (superoxide donor) dosed to generate superoxide levels in the physiologically relevant range produced by activated NADPH oxidase[52, 53]. Sampling was performed at 0, 1, 3 and 6hrs from separated cells and supernatants (2,000*g*, 10min, 4°C). Catabolism of [1,2,3-^13^C_3_]glucose generates distinct lactate isotopologues, namely ^13^C_2_ (M+2) and ^13^C_3_ (M+3), depending on whether glucose oxidation preferentially occurs through the pentose phosphate pathway (PPP) or the Embden-Meyerhof-Parnas (EMP) glycolytic pathway, respectively.

Samples were prepared for UHPLC-MS metabolomics, as described[50] (also, see S1 Extended Methods). The Q Exactive mass spectrometer (Thermo, Bremen, Germany) was operated in Full MS mode (2μscans) at 70,000 resolution in the 60-900m/z range, 4kV spray voltage, 15 sheath gas and 5 auxiliary gas, in positive and negative ion modes (separate runs). Metabolite assignments and isotopologue distributions were determined using the software Maven (Princeton, NJ)[54] and assignments confirmed against 700 standards (IROATech, Sigma Aldrich, St. Louis, MO).

Partial-Least Square Discriminant Analysis (PLS-DA), hierarchical clustering analysis (Pearson correlation, complete linkage) and heat maps were plotted with MetaboAnalyst 4.0[55] and GENE E (Broad Institute, Cambridge, MA).

### In vivo analysis of RBC adhesion, RBC velocity, and Hb saturation

Nude mice (n = 16; *Foxn1*^*nu*^; Jackson Labs, strain 002019) were anesthetized (isoflurane) and fitted with a dorsal window chamber, as described[56]. After recovery (1-3d), mice were anesthetized (isoflurane), placed upon a temperature-controlled platform and inspected to verify intact microcirculation. PKH-26 labeled RBCs (10μL/g body weight) were infused over 10sec and label movement was tracked by intravital microscopy (5X, Zeiss Axioobserver Z1, Zeiss Zen software). Videos (transmission and red fluorescent channel) and still images of the microvasculature were captured for each subject and analyzed offline for RBC adhesion, RBC velocity, and Hb saturation (ImageJ, NIH)(MatLab R2017b, Mathworks)[57].

Adhesion was measured by time-averaging a ~60sec sequence, yielding a composite image in which adherent cells appeared as distinct fluorescent entities, while moving cells blurr. Individual adherent RBCs were then hand-counted. Adhesion values for each subject were calculated and presented as percent adherent cells relative to total cells present in a single frame.

To measure RBC velocity, five venules of various sizes were randomly selected from each subject’s video. Individual RBCs were selected from these venules, then tracked through 5–10 frames of movement. Because individual RBC velocities through different venules is non-uniform, two cells were selected from a particular venule and their velocities through a shared segment of that venule were averaged. Venule diameter was measured perpendicular to the mid-point of the travel path. Each two-cell velocity average was then normalized to the inverse square of the radius of the venule segment through which the cells passed. This provided five normalized RBC velocities per subject, which were then averaged to yield a single (n = 1) average radius^2^-normalized RBC velocity per subject.

To measure Hb saturation, still images of each subject were taken at a range of wavelengths and processed (Matlab) to generate a Hb saturation heat map of the region’s venules[56]. Five segments corresponding to those used for the velocity analysis were selected for each subject. The Hb saturation values of these segments were averaged to give a single final averaged Hb saturation percentage value for each subject.

### Statistical analyses

Results are presented as mean ± standard error of the mean (SEM). Column statistics were performed to determine data distribution normality. Where appropriate, groups were compared by t-test (Student’s or Wilcoxon rank) or repeated measures analysis of variance (RMANOVA; Bonferroni’s posthoc test) (PRISM, GraphPad Inc.; La Jolla, CA).

## Results

### RBC glycerolization

We initially attempted glycerolizing RBCs in one-step, adding 60% v/v buffer/glycerol solution to pelleted RBCs (40% v/v, glycerol final). The effect of temperature on this process was significant, with warmer glycerol resulting in less RBC lysis (Fig 1B). This method resulted in lysis (prior to freezing) of ~10%, which we considered unacceptable. We then optimized a two-step method (similar to that reported[28, 38, 39]). We found the order of component addition to strongly influence hemolysis; adding buffer (PBS 300μl) and 300μl buffer/glycerol stock (60% v/v glycerol in starting buffer solution) to the tube first, followed by RBCs (300μl), resulted in lowest lysis (Fig 1C). We also tested thawing temperature (37°C *vs* 50°C) as well as different cryopreservative tubes (Biocision TruCool cryogenic vials #BCS-2502 and 2504, Larkspur, CA; Corning cryogenic vials # 430487, NY; ThermoFisher Scientific Abgene storage tubes # AB1411, Waltham, MA) and tube sizes (TruCool, Biocision, Larkspur, CA # BCS-2502, #BCS-2504), but no significant differences were observed (not shown). In comparison to standard glycerol cryopreservation[38, 39], our adapted procedure (Fig 1A) resulted in similar % lysis (1.18% ± 0.2% vs 0.88% ± 0.1%, standard vs adapted, respectively; Fig 1D), following sample freeze/thaw (-80°C overnight in a Coolcell and next-day thawing (37°C, 2min)); of note, sample processing time was reduced by 40% (from 25 to 15min).

### RBC deglycerolization

With the standard deglycerolization method[28, 39] (Fig 2A), we observed total cumulative lysis of 13.0% ± 1.5%, from thawing to complete glycerol removal (Fig 2C). Consequently, to reduce lysis, we slowed the rate at which osmolality was lowered during deglycerolization (Fig 2B). With this adapted method, total cumulative lysis fell to 8.4% ± 0.3% (p<0.05, ~ 35% reduction) (Fig 2C). Glycerol concentration was measured to ensure full CPA removal (>99.9% for both the standard and adapted deglycerolization protocols, Fig 2D).

### Standard morphological imaging (SEM) and measurement (CBC)

**Fig 3:**
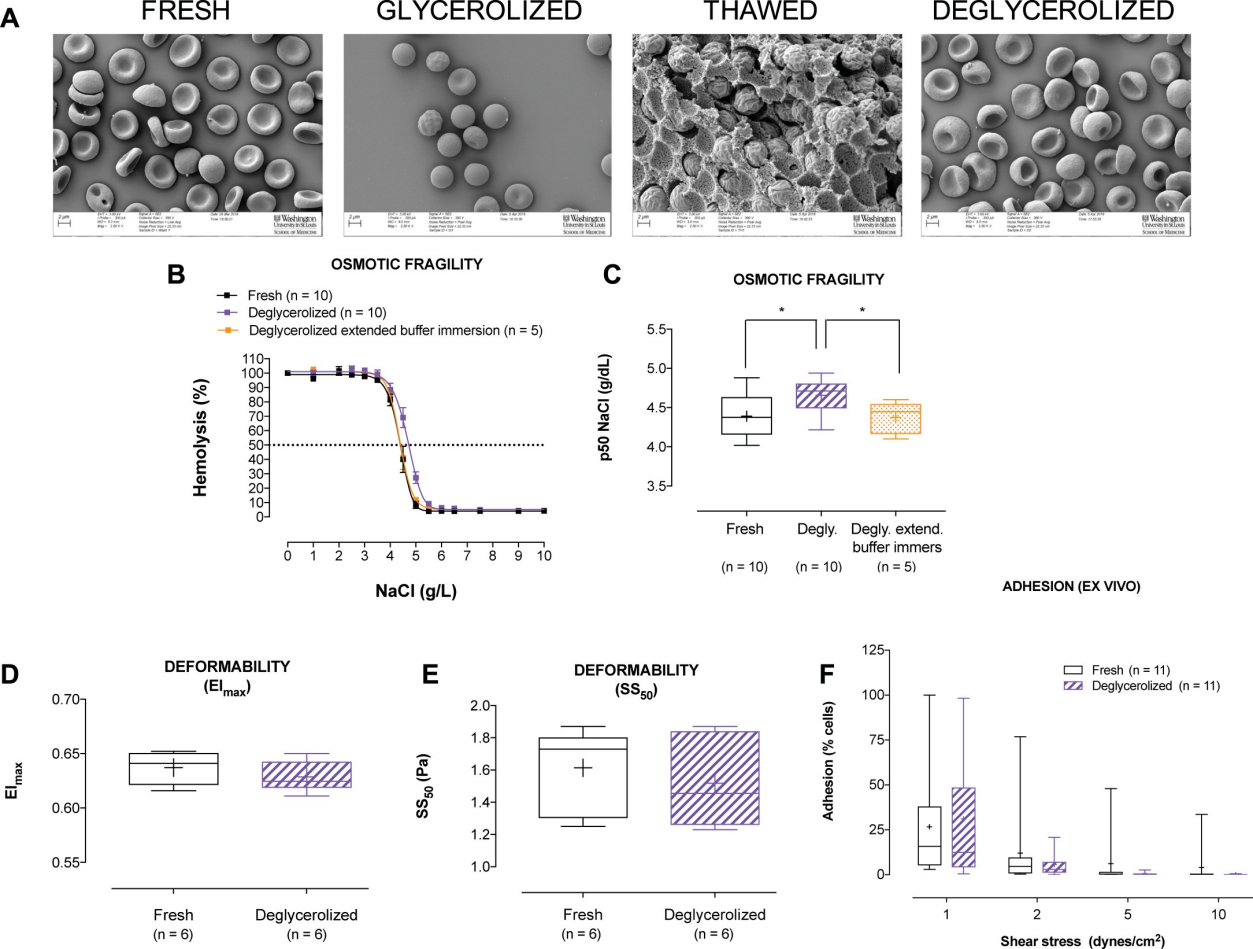
RBC morphological imaging (scanning electron microscope; SEM) and comparison of osmotic fragility, deformability, and *ex vivo* adhesion in matched fresh and deglycerolized RBCs. **(A)** Illustration of the characteristic biconcave disc morphology of fresh RBCs. Following glycerol addition, the majority of RBCs become swollen and spherocytic. After thawing, RBCs remain encapsulated in a glycerol matrix. Following deglycerolization RBCs return to their original morphology. **(B)** NaCl induced osmotic lysis in matched fresh, deglycerolized, and deglycerolized RBCs exposed to a prolonged buffer incubation (n = 5 or 10 individual donors, shown on graph). NaCl induced osmotic lysis increased immediately following deglycerolization, but returned to the original fresh phenotype following prolonged incubation (1 hour) of RBCs in the final deglycerolization buffer. **(C)** Half maximal effective concentration (EC_50_) increased immediately following deglycerolization, in comparison to fresh RBC samples (p < 0.05). However, prolonged incubation (1 hour) of RBCs in the final deglycerolization buffer at 37°C restored the original fresh RBC phenotype (n = 5 or 10 individual donors, shown on graph). **(D)** Shear induced maximal RBC deformability (Elongation Index maximum; EI_max_) was not different between matched fresh and deglycerolized RBCs (exposed to the prolonged buffer immersion) (n = ±6 individual donors). **(E)** The shear stress (Pa) at which RBC demonstrated 50% elongation (SS_50_) was also not different between matched fresh and deglycerolized RBCs (exposed to the prolonged buffer immersion) (n = 6 individual donors). **(F)** RBC adhesivity to endothelium was determined *ex vivo*. A trend was observed toward a decrease in RBC adhesivity in the deglycerolized RBCs, across all sheer stresses > 1 dyne/cm^2^, that did not reach statistical significance.

SEM images were acquired at each stage of sample assessment (Fig 3A and S1 Fig). Fresh RBCs demonstrated the characteristic bi-concave disc shape, with minimal morphological variance. Following glycerolization, the majority of cells appeared spherocytic, as reflected in the low morphological index (Im = 40.6 ± 2.1). After thawing, RBCs were observed to be embedded in a highly structured cryopreservation matrix, with almost all cells demonstrating a crenated sphere morphology (Im = 20.1 ± 0.1). Following full deglycerolization, cell morphology was indistinguishable from that for fresh RBCs, with no significant difference in morphological index (Im = 97.1 ± 1.1 vs 99.1 ± 0.2, respectively; paired t-test p = 0.26).

RBC morphometric indices complemented the qualitative SEM images. Compared to fresh RBCs, glycerolization increased MCV (91.2 ± 2.1fL vs 109.7 ± 1.7fL; p<0.05; S1 Fig), without altering MCH (S1 Fig this was corroborated by MCHC decrease (32.2 ± 0.3g/dL vs 26.7 ± 0.3g/dL; p<0.05; S1 Fig), corresponding to observed spherocytic morphology (SEM). Glycerolization also increased RDW, compared to fresh RBCs (34.5 ±0.8% vs 13 ± 0.3%, respectively; p<0.05; S1 Fig). Following full deglycerolization, all parameters returned to levels observed for fresh RBCs.

### *In vitro* physiologic measurements

#### Cell volume regulation (osmotic fragility)

When measured immediately following deglycerolization, storage in glycerol and full deglycerolization increased RBC osmotic fragility (i.e., cells lysed at a higher tonicity [in NaCl solution] in comparison to fresh RBCs); the half-maximal effective NaCl concentration [EC_50_] increased (from 4.39 ± 0.09g/L to 4.66 ± 0.07g/L, p<0.05; Fig 3B and 3C). However, resting RBCs in the last deglycerolization buffer (1h, 37°C) restored osmotic resilience to levels observed in fresh RBCs (4.39 ± 0.09g/L to 4.37 ± 0.09g/L; Fig 3B and 3C).

#### Deformability

Deformability of deglycerolized and fresh RBCs was identical across a physiologically relevant range of shear stress (Fig 3D). The shear stress at which RBCs demonstrated 50% elongation (SS_50_) was did not differ between fresh and deglycerolized RBCs (following prolonged buffer immersion) (Fig 3E).

#### Adhesion

A trend was observed toward decreased *ex vivo* RBC adhesivity in deglycerolized compared to fresh RBCs, across all sheer stresses >1dyne/cm^2^; this did not reach statistical significance (Fig 3F).

#### O_2_ affinity

**Fig 4:**
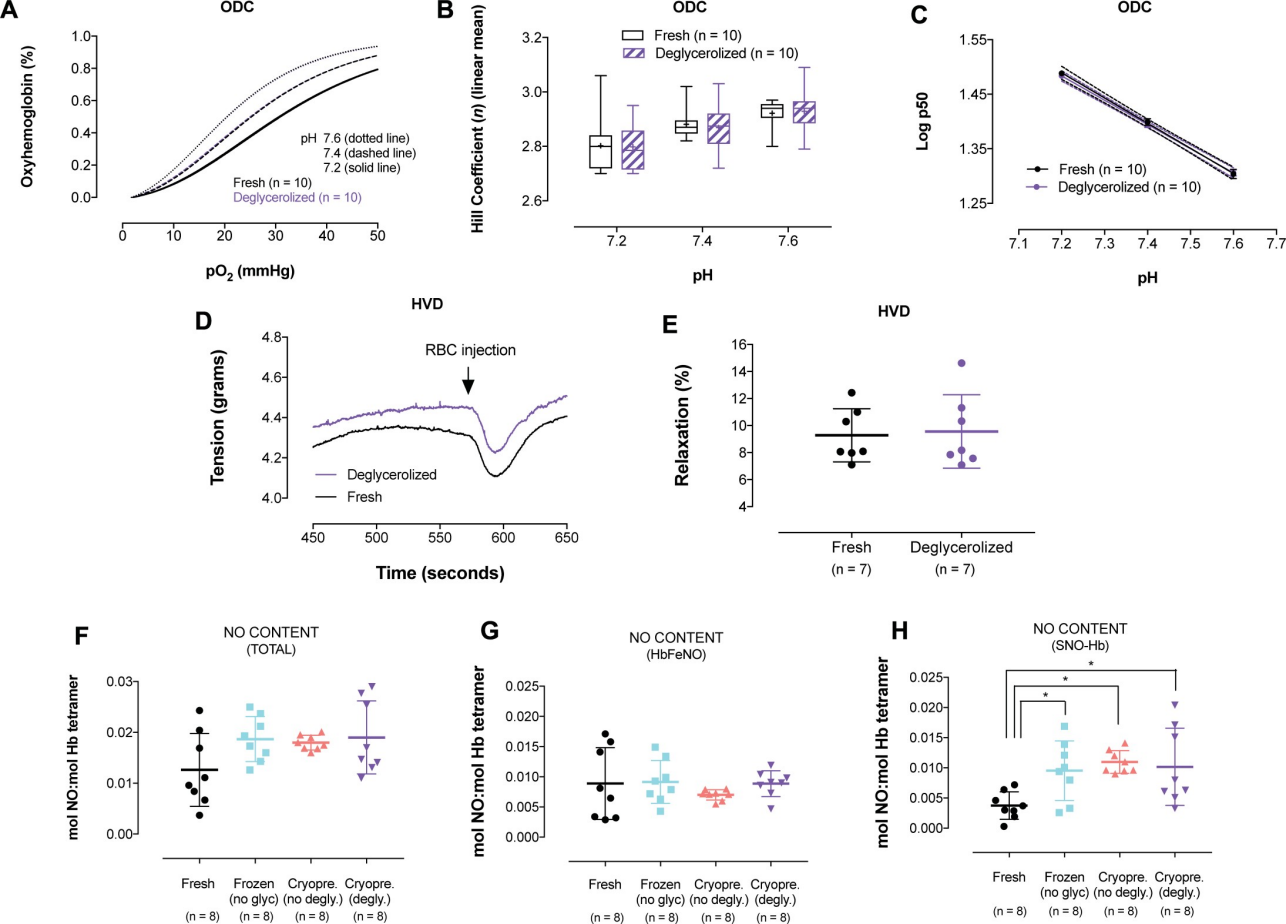
Comparison of O_2_ dissociation curves (ODCs) and RBC hypoxic vasodilatory response (HVD) from matched fresh and deglycerolized RBCs, and NO content of matched fresh RBCs, frozen RBCs (without glycerol), cryopreserved RBCs (without glycerol removal), and cryopreserved RBCs (deglycerolized), measured by photolysis:chemiluminescence. **(A)** Raw ODC curves from matched fresh and deglycerolized RBCs in BIS TRIS buffer (BIS TRIS 50mM, NaCl, 100mM), pH to 7.2, 7.4, and 7.6. Inset is the whole ODC curve (n = 10 individual donors). No difference was observed between fresh and deglycerolized RBCs. **(B)** Hill coefficient, a measure of cooperativity, was not different between matched fresh and deglycerolized RBC samples across the 3 different pH values. **(C)** Bohr plot demonstrates no change between matched fresh and deglycerolized RBC samples. **(D)** Representative single traces (from rings demonstrating similar PE constriction responses) of the relaxation responses from fresh *vs* deglycerolized RBCs under hypoxia (~ 1% O_2_)—Arrow indicates injection point for RBCs. **(E)** Relaxation responses of fresh and deglycerolized RBCs (as a percentage of maximal constriction) are not different in a hypoxic organ chamber bioassay of endothelial intact rabbit aortic rings. **(F)** Total RBC NO content was not significantly different between any of the conditions, although freezing appeared to slightly augment NO levels. **(G)** Iron nitrosyl hemoglobin (HbFeNO) content remained unchanged in all conditions. **(H)** S-nitrosohemoglobin (SNO-Hb) significantly increased upon freezing, independent of glycerol presence (p < 0.05). Data reported as NO:Hb (tetramer basis).

No difference was observed in the O_2_-hemoglobin dissociation curves from matched fresh and deglycerolized RBC samples (BIS TRIS 50mM, NaCl, 100mM) (Fig 4A), nor in p50, cooperativity (Fig 4B) and Bohr effect (Fig 4C).

#### RBC-based hypoxic vasodilatory response

In an organ-chamber bioassay of endothelium-intact rabbit aortic rings held at low O_2_ tension (~1% O_2_), matched fresh and deglycerolized RBCs demonstrated similar vasodilation (Fig 4E). Single representative traces were plotted to visually demonstrate the relaxation response (Fig 4D).

#### RBC NO content

Photolysis-chemiluminescence was used to measure RBC hemoglobin NO content in matched: fresh, frozen (without glycerol) and cryopreserved RBCs (without/with glycerol removal). Total Hb NO content was not significantly different between any of the conditions, although freezing (regardless of glycerol addition) appeared to slightly augment NO levels (Fig 4F). Iron nitrosyl hemoglobin (HbFeNO) content remained unchanged in all conditions (Fig 4G). However, S-nitrosohemoglobin (SNO-Hb) levels increased upon sample freezing (Fig 4H), independent of glycerol presence or removal (fresh: 0.0038 ± 0.001; frozen: 0.0095 ± 0.002; cryopreserved w/o deglycerolization: 0.011 ± 0.001; cryopreserved, deglycerolized: 0.010 ± 0.002; expressed as NO:Hb molar ratio; tetramer basis, for all; p<0.05).

### Biochemical analysis

#### Metabolomics–steady state phenotypes and metabolic fluxes in response to oxidant stress

**Fig 5:**
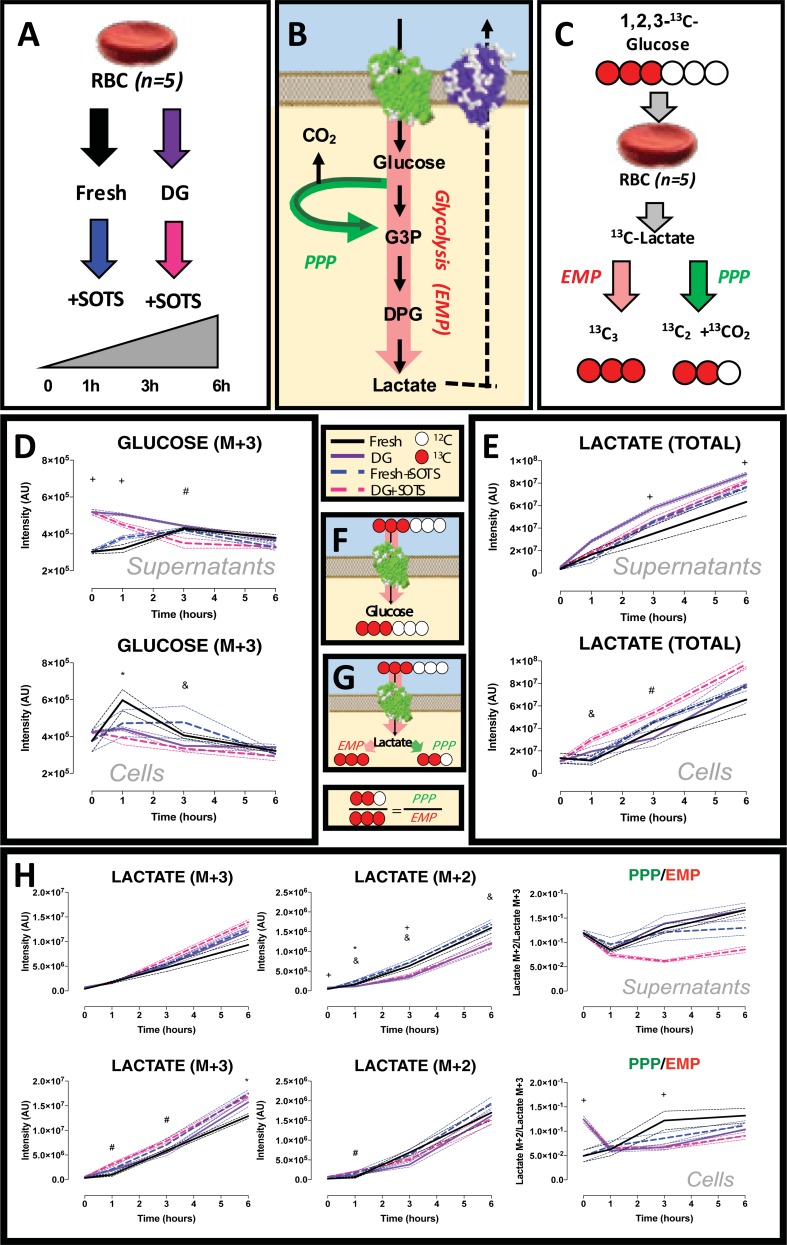
Comparison of metabolomic steady state phenotypes and metabolic fluxes in response to oxidant stress in fresh and matched deglycerolized RBCs. **(A)** Tracing experiments were performed by incubating RBCs with [1,2,3-^13^C_3_]glucose, a heavy substrate that can be metabolized in RBCs through **(B)** the Embden-Meyerhof-Parnas (EMP) glycolytic pathway (generating isotopologue M+3) or through the pentose phosphate pathway (PPP), generating isotopologue M+2 of lactate, **(C)** owing to the release of the first carbon atom of heavy glucose in the form of ^13^CO_2_ at the oxidative phase reactions of the PPP. **(F)** Schematic overview of glucose uptake and consumption in RBC supernatants and cells, in fresh, or deglycerolized RBCs, either untreated, or treated with the superoxide generator (SOTS-1), **(D)** demonstrating in both RBCs and media similar glucose consumption rates. **(E)** Total lactate, i.e., sum of lactate isotopologues (unlabeled M+0, M+3 generated via glycolysis, or M+2 generated via the PPP), in fresh and DG RBCs, either untreated or treated with the superoxide generator (SOTS-1), demonstrating in both RBCs and media similar lactate accumulation. **(G and H)** Schematic overview of expected isotopologues and relative ratios of lactate, from glycolysis (EMP, M+3 isotopologue) or PPP (M+2) and relative ratios in fresh and DG RBCs, either untreated, or treated with the supoxide generator SOTS-1. **(H)** Fresh RBCs were characterized by significantly higher levels of supernatant–but not intracellular—M+2 lactate. Conversely, deglycerolized RBCs were characterized by faster glycolysis than fresh RBCs. Lower lactate M+2 export in deglycerolized RBCs following SOTS-1 was observed, while intracellular glycolysis to pentose phosphate pathway ratios were comparable between fresh and deglycerolized RBCs following treatment with SOTS-1. Continuous lines indicate median, while dashed lighter lines are representative of interquartile ranges, according to the color legend left. Symbols indicate p<0.05 **+** = fresh vs DG; **&** = fresh + SOTS vs DG + SOTS; * = fresh vs fresh + SOTS; **#** = DG vs DG + SOTS.

UHPLC-MS metabolomics were performed to compare phenotypes of fresh vs cryopreserved and deglycerolized RBCs (Fig 5A–5H). Analyses of both RBCs and media indicated similar glucose consumption rates (Fig 5D and 5F) and total lactate generation (Fig 5E and 5G) in both groups over a 6h time course. Oxidative loading (SOTS-1) was employed to unveil more subtle metabolic differences between fresh and deglycerolized RBCs, by extending the dynamic range for metabolite flux. Supplementation to the media of [1,2,3-^13^C_3_]glucose was used to determine the rate of lactate isotopologue enrichment via glycolysis (M+3) or the pentose phosphate pathway (M+2). Results indicated that fresh RBCs were characterized by significantly higher levels of supernatant–but not intracellular—M+2 lactate (Fig 5H). Conversely, deglycerolized RBCs were characterized by faster glycolysis than fresh RBCs (Fig 5H). Even though lower lactate M+2 export in deglycerolized RBCs following SOTS-1 was observed, intracellular glycolysis to pentose phosphate pathway ratios were comparable between fresh and deglycerolized RBCs following treatment with SOTS-1 (Fig 5H). Deglycerolized RBCs were also characterized by lower levels of oxidized glutathione (GSSG) and higher reduced to oxidized glutathione ratios (GSH/GSSG) in comparison to fresh RBCs treated with SOTS-1, suggestive of decreased oxidative damage upon oxidative loading, in comparison to SOTS-1-treated fresh RBCs (not shown).

**Fig 6:**
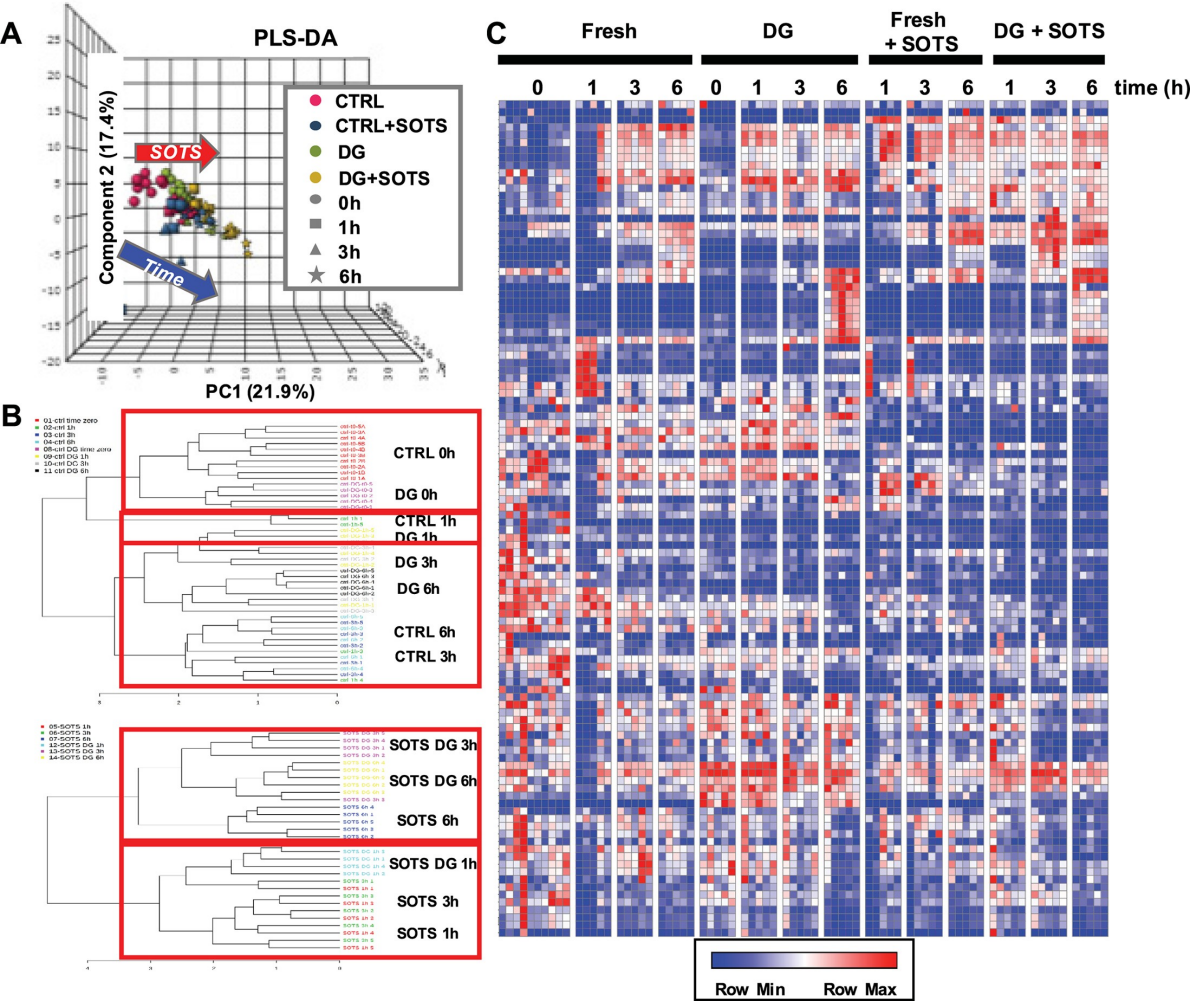
Unsupervised analysis of metabolomics data via PLS-DA, with dendrograms for time course hierarchical clustering and heat maps, visually indicating a time course and SOTS dependency of the metabolic phenotypes observed in fresh RBC and matched DG RBCs. **(A)** The impact of time and SOTS treatment informed sample clustering along principal components 1 and 2 (explaining 21.9 and 17.4% of the total variance, respectively). Both **(A)** PLS-DA and **(B)** hierarchical clustering analyses confirmed that control and DG groups fell into the same clusters at the tested time points, both in the absence and presence of SOTS. **(C)** Heat maps visually indicate the time course and SOTS dependency of the metabolic phenotypes observed both in fresh RBC and DG groups.

Considering the minor differences noted in (above) tracing exeperiments, to further compare the metabolic phenotypes of fresh and cryopreserved RBCs, we performed unsupervised analyses of metabolomics data via PLS-DA (Fig 6A) and hierarchical clustering. Dendrograms for time course data from control vs DG +/- SOTS-1 are shown in Fig 6B. The impact of time and oxidative loading (SOTS-1) informed sample clustering along principal components 1 and 2 (explaining 21.9 and 17.4% of the total variance, respectively). Both PLS-DA and hierarchical clustering analyses confirmed that control and DG groups fell into identical clusters at the tested time points, both in the absence and presence of SOTS (Fig 6A and 6B). Results are further shown in the form of heat maps (Fig 6C and S2 Fig), visually indicating a time course and SOTS-1 dependency of the metabolic phenotypes observed both in control and DG groups. The only notable exception was glycerol-phosphate, a product of glycerol metabolism, observed to be significantly higher in DG RBCs at 0h time point and declining to 0h control levels just after 6h.

### In vivo RBC analysis

#### RBC response to transfusion in nude mice (mapped hemoglobin O_2_ saturation, RBC velocity, and RBC adhesion)

**Fig 7:**
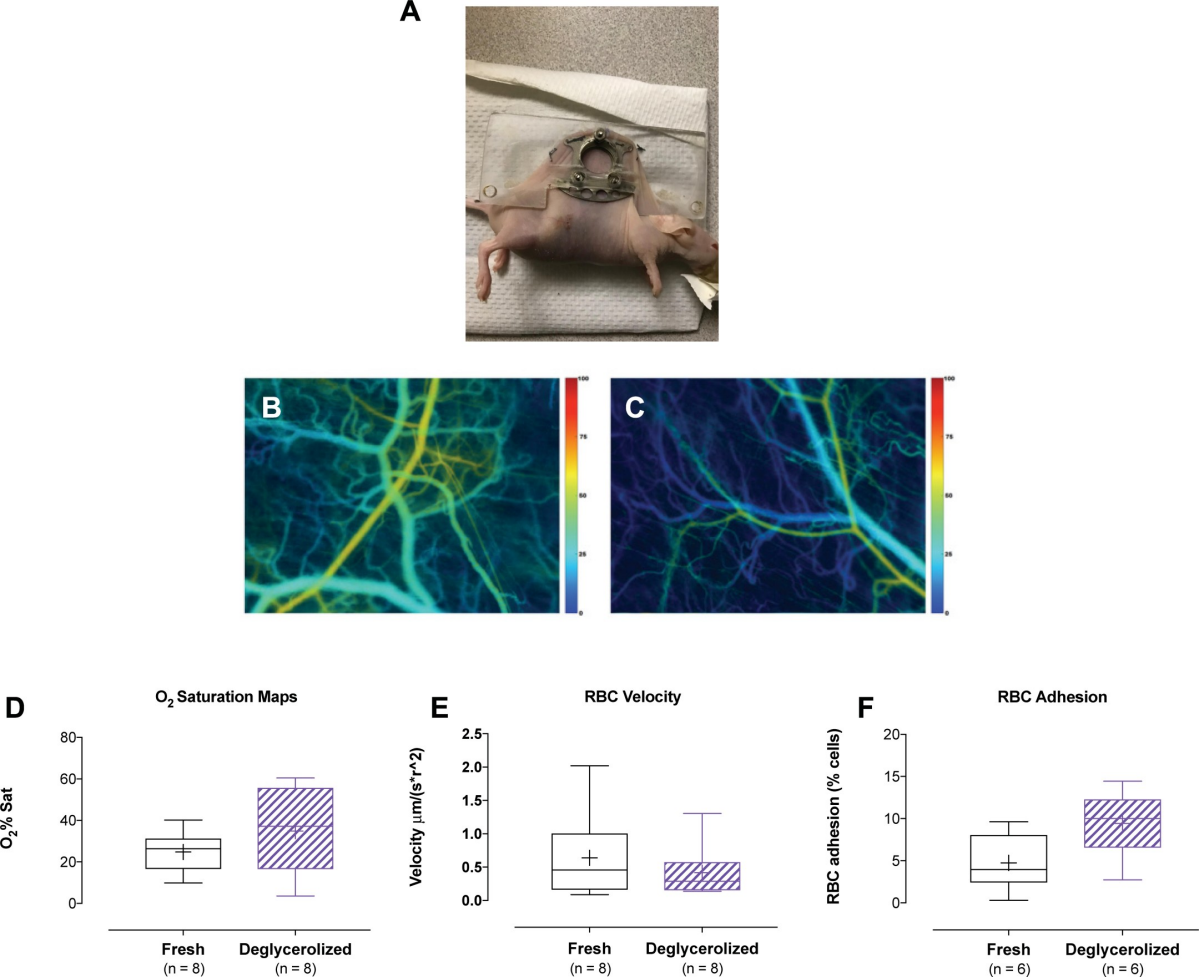
Dorsal window chamber set up for the assessment of *in vivo* hemoglobin O_2_ saturation, RBC velocity and vascular adherence of transfused fresh or matched deglycerolized RBCs. **(A)** Image of the dorsal window chamber set up. **(B** and **C)** No difference was observed in mapped hemoglobin O_2_ saturation, or **(D)** RBC velocity in the dorsal skinfold window chamber model, between the transfusion of fresh or deglycerolized RBCs. **(E)** A modest, but statistically insignificant difference was observed on *in vivo* adhesivity of transfused RBCs between fresh and deglycerolized RBCs from the same donors.

Post-transfusion function of human RBCs was assessed in a nude mouse model (Fig 7A), with comparison made between fresh or deglycerolized RBCs. Labeled human RBCs were transfused into healthy nude mice in volumes mimicking a 2-unit transfusion in an adult human. As described[58, 59], control fresh healthy human RBCs did not alter blood hemoglobin O_2_ saturation maps. There was also no effect on (transfused) RBC velocity. Adhesion of control transfused human RBCs *in vivo* was low, and adhesion of transfused DG RBCs did not differ significantly.

We found that the cryopreservation protocol did not alter mapped hemoglobin O_2_ saturation in a dorsal skinfold window chamber model either qualitatively or in quantitative Hb saturation analysis (Fig 7B and 7C), nor was RBC velocity altered (Fig 7D). There was a modest but statistically insignificant effect of cryopreservation on *in vivo* adhesivity of transfused RBCs (Fig 7E), as compared to that of fresh human RBCs from the same donors.

## Discussion

Here, we report a RBC cryopreservation protocol optimized to satisfy requirements particular to RBC research. This protocol improved glycerolization efficiency (~40% time reduction to achieve 40% v/v, glycerol final) and was equivalent to the standard method[28, 38, 39] in terms of RBC recovery following freezing (-80°C) and thawing (37°C). Of note, following full glycerol removal (>99.9%), our modified procedure reduced total cumulative lysis by ~35%, in comparison to the standard method. These improvements minimize the burden of initial sample processing, render cryopreservation protocols more feasible for clinical research staff, improve RBC recovery following cryopreservation and enable analyses of vulnerable RBC sub-populations that might otherwise be lost. Our modified methodology resulted in a post-cryopreservation/deglycerolization RBC phenotype that was indistinguishable from that of fresh RBCs, with regard to physical RBC parameters (morphology, volume, and density), osmotic fragility, deformability, endothelial adhesivity, O_2_ affinity, vasoregulation, metabolomics, and flow dynamics.

In the process of inter-laboratory method validation, we noted that sample handling during both the glycerolization and deglycerolization procedures influenced RBC lysis amount. Others[34] have also reported that RBC sample processing contributes more to cryostored RBC quality than does storage duration. Specificially, we determined that when adding packed RBCs to glycerolizing solutions, immediate gentle sample inversion (to fully suspend cells) was necessary to prevent RBC clumping and greater post-thaw lysis. Similarly, throughout the deglycerolization procedure, lysis was increased by excessive agitation or tube plucking/tapping to accelerate RBC re-suspension. Ultimately, simple rotation of the tube on its axis (to mix the sample) proved optimal. We did not address the effect of storage duration on RBC recovery or phenotype fidelity, as all samples were analyzed and measured within a month of freezing.

Our cryopreservation protocol was specifically developed for maximal recovery of human RBCs. It should be noted that plasma osmolality[60–62] as well as RBC size and volume (and subsequently surface area to volume ratios)[63–68] differ widely across species commonly used in RBC research, all of which may contribute to differing tolerance to osmotic and mechanical stress. It is thus likely that species-specific adjustments to this cryopreservation protocol may be needed to attain similar results. Additionally, even with human RBCs, we still observed 8% lysis following cryopreservation. We speculate that the cells lost may represent cells or cell sub-population(s) least resilient to osmotic or mechanical stress, suggesting possible need for additional method validation prior to study of subjects with such pathologies. With this in mind, while we focused on glycerol as our CPA due to its lack of toxicity and extensive literature on its clinical use, other cryoprotectants are available (both cell-penetrating and non-cell penetrating) that might better address this issue.

The finding that there was a small degree of baseline adhesion of control transfused human RBCs is in agreement with prior published findings[58, 59]. It is possible that the slightly larger size of human red cells may lead to pseudostatic appearance in mouse capillaries, where some fraction of the transfused RBCs might require a slightly longer transit time. In addition, there may be infrequent, random adhesion of even murine RBCs to murine (or human to human) endothelium *in vivo*. The adapted cryopreservation technique did not increase RBC adhesivity.

Unsupervised analyses of metabolomics data confirmed similarity of metabolic phenotypes of fresh and deglycerolized RBCs, both at rest and under oxidant loading conditions, affirming the conclusion that our research-optimized procedure for RBC cryopreservation and deglycerolization in 40% v/v glycerol final does not significantly impact RBC phenotype (compared to fresh cells).

## Supporting information

S1 Extended Methods (DOC)

S1 Fig (DOC)

S2 Fig (DOC)

## References

[1] Smith AU. Prevention of haemolysis during freezing and thawing of red blood-cells. Lancet. 1950;2(6644):910–1. . 14795743 10.1016/s0140-6736(50)91861-7

[2] Lecak J, Scott K, Young C, Hannon J, Acker JP. Evaluation of red blood cells stored at -80 degrees C in excess of 10 years. Transfusion. 2004;44(9):1306–13. 10.1111/j.1537-2995.2004.03271.x . 15318853

[3] Valeri CR, Ragno G, Pivacek LE, Cassidy GP, Srey R, Hansson-Wicher M, et al An experiment with glycerol-frozen red blood cells stored at -80 degrees C for up to 37 years. Vox sanguinis. 2000;79(3):168–74. 31236. 10.1159/000031236 . 11111236

[4] Pegg DE. Principles of Cryopreservation In: Wolkers WF, Oldenhof H., editor. Cryopreservation and freeze-drying protocols Methods in Molecular Biology (Methods and Protocols). 1257 New York, NY: Springer; 2015 p. 3–19. 10.1007/978-1-4939-2193-5_1 25428001

[5] Gao D, Critser JK. Mechanisms of cryoinjury in living cells. ILAR J. 2000;41(4):187–96. . 11123179 10.1093/ilar.41.4.187

[6] Mazur P. Kinetics of Water Loss from Cells at Subzero Temperatures and the Likelihood of Intracellular Freezing. J Gen Physiol. 1963;47:347–69. 14085017 10.1085/jgp.47.2.347 PMC2195343

[7] Mazur P. Stopping biological time. The freezing of living cells. Annals of the New York Academy of Sciences. 1988;541:514–31. . 3058000 10.1111/j.1749-6632.1988.tb22288.x

[8] Lovelock JE. The haemolysis of human red blood-cells by freezing and thawing. Biochimica et biophysica acta. 1953;10(3):414–26. . 13058999 10.1016/0006-3002(53)90273-x

[9] Lovelock JE. The mechanism of the protective action of glycerol against haemolysis by freezing and thawing. Biochimica et biophysica acta. 1953;11(1):28–36. . 13066452 10.1016/0006-3002(53)90005-5

[10] Leibo SP. Fundamental cryobiology of mouse ova and embryos. Ciba Found Symp. 1977;(52):69–96. . 244402 10.1002/9780470720332.ch5

[11] Fahy GM, Wowk B. Principles of Cryopreservation by Vitrification In: Wolkers W, Oldenhof H., editor. Cryopreservation and Freeze-Drying Protocols Methods in Molecular Biology (Methods and Protocols). 1257 New York, NY: Springer; 2015 p. 21–82. 10.1007/978-1-4939-2193-5_2 25428002

[12] Hunter FR. Facilitated diffusion in pigeon erythrocytes. The American journal of physiology. 1970;218(6):1765–72. 10.1152/ajplegacy.1970.218.6.1765 . 5446311

[13] Merryman HT. Freezing and vitrification of red cells, recollections and predictions In: Smit Sibinga CT, Cash J.D., editor. Transfusion Medicine: Quo Vadis? What has been Achieved, What is to be Expected Developments in Hematology and Immunology. Boston, MA: Springer; 2001 p. 69–85.

[14] Lagerberg JW. Cryopreservation of red blood cells In: Wolkers WF, Oldenhof H., editor. Cryopreservation and freeze-drying protocols Methods in Molecular Biology (Methods and Protocols). 1257 New York, NY: Springer; 2015 p. 353–67. 10.1007/978-1-4939-2193-5_17 25428017

[15] Tullis JL, Gibson J.G., Sproul M.T., Tinch R.J., Baudanze P. Advantages of the high glycerol mechanical systems for red cell preservation: A 10 year study of stability and yield In: Spielmann W, Seidl S., editor. Modern Problems of Blood Preservation. Stuttgart: Fischer; 1970 p. 161–7.

[16] Valeri CR. Recent advances in techniques for freezing red cells. CRC Crit Rev Clin Lab Sci. 1970;1(3):381–425. . 4999621 10.3109/10408367009027949

[17] Krijnen HW, De Wit JJ, Kuivenhoven AC, Loos JA, Prins HK. Glycerol Treated Human Red Cells Frozen with Liquid Nitrogen. Vox sanguinis. 1964;9:559–72. . 14221593 10.1111/j.1423-0410.1964.tb03326.x

[18] Rowe AW, Eyster E, Kellner A. Liquid nitrogen preservation of red blood cells for transfusion; a low glycerol-rapid freeze procedure. Cryobiology. 1968;5(2):119–28. . 5717951 10.1016/s0011-2240(68)80154-3

[19] Valeri CR, Ragno G., Pivacek L.E., Srey R., Hess J.R., Lippert L.E., et al A multicenter study of in vitro and in vivo values in human RBCs frozen with 40-percent (wt/vol) glycerol and stored after deglycerolization for 15 days at 4 degrees C in AS-3: assessment of RBC processing in the ACP 215. Transfusion. 2001;41(7):933–9. 11452163 10.1046/j.1537-2995.2001.41070933.x

[20] Valeri CR, Srey R, Tilahun D, Ragno G. The in vitro quality of red blood cells frozen with 40 percent (wt/vol) glycerol at -80 degrees C for 14 years, deglycerolized with the Haemonetics ACP 215, and stored at 4 degrees C in additive solution-1 or additive solution-3 for up to 3 weeks. Transfusion. 2004;44(7):990–5. 10.1111/j.1537-2995.2004.04001.x . 15225238

[21] Valeri CR, Pivacek L.E., Gray A.D., Cassidy G.P., Leavy M.E., Dennis R.C., et al The safety and therapeutic effectiveness of human red cells stored at -80 degrees C for as long as 21 years. Transfusion. 1989;29(5):429–37. . 2734823 10.1046/j.1537-2995.1989.29589284145.x

[22] Valeri CR, Ragno G., Pivacek L, O’Neill E.M. In vivo survival of apheresis RBCs, frozen with 40-percent (wt/vol) glycerol, deglycerolized in the ACP 215, and stored at 4°C in AS-3 for up to 21 days. Transfusion. 2001;41:928–32. 11452162 10.1046/j.1537-2995.2001.41070928.x

[23] Lelkens CCM, Noorman F., Koning J.G., Truijens-de Lange R., Stekkinger P.S., Bakker J.C., et al Stability after thawing of RBCs frozen with the high- and low-glycerol method. Transfusion. 2003;43(2):157–64. 12559010 10.1046/j.1537-2995.2003.00293.x

[24] Capicciotti CJ, Kurach J.D.R., Turner T.R., Mancini R.S., Acker J.P., Ben R.N. Small Molecule Ice Recrystallization Inhibitors Enable Freezing of Human Red Blood Cells with Reduced Glycerol Concentrations. Scientific reports. 2015;5. 10.1038/srep09692 PMC4389209 25851700

[25] Sen AK, A. Comparative study of automated cryopreservation of red blood cells. Med J Armed Forces India. 2013;69(4):345–50. 10.1016/j.mjafi.2013.06.005 24600141 PMC3862465

[26] Valeri CR, Pivacek L.E., Cassidy G.P., Ragno G. Posttransfusion survival (24-hour) and hemolysis of previously frozen, deglycerolized RBCs after storage at 4 degrees C for up to 14 days in sodium chloride alone or sodium chloride supplemented with additive solutions. Transfusion. 2000;40(11):1337–40. 11099661 10.1046/j.1537-2995.2000.40111337.x

[27] Moore GL, Ledford M.E., Mathewson P.J., Hankins D.J., Shah S.B. Post-thaw storage at 4 degrees C of previously frozen red cells with retention of 2,3-DPG. Vox sanguinis. 1987;53(1):15–8. 3660765 10.1111/j.1423-0410.1987.tb04906.x

[28] Meryman HT, Hornblower M. A simplified procedure for deglycerolizing red blood cells frozen in a high glycerol concentration. Transfusion. 1977;17(5):438–42. . 910260 10.1046/j.1537-2995.1977.17578014580.x

[29] Hess JR. Red cell freezing and its impact on the supply chain. Transfusion medicine. 2004;14(1):1–8. 10.1111/j.0958-7578.2004.00472.x . 15043586

[30] Scott KL, Lecak J, Acker JP. Biopreservation of red blood cells: past, present, and future. Transfus Med Rev. 2005;19(2):127–42. . 15852241 10.1016/j.tmrv.2004.11.004

[31] Huggins CE. Reversible agglomeration—a practical method for removal of glycerol from frozen blood In: Spielmann W, Seidl S., editor. Modern Problems of Blood Preservation. Stuttgart: Fischer; 1970 p. 138–55.

[32] Standards for Blood Banks and Transfusion Services. 29th ed. Bethesda, Md: American Association of Blood Banks (AABB); 2014.

[33] Pallotta V, D'Amici GM, D'Alessandro A, Rossetti R, Zolla L. Red blood cell processing for cryopreservation: from fresh blood to deglycerolization. Blood cells, molecules & diseases. 2012;48(4):226–32. 10.1016/j.bcmd.2012.02.004 . 22424604

[34] Henkelman S, Lagerberg JW, Graaff R, Rakhorst G, Van Oeveren W. The effects of cryopreservation on red blood cell rheologic properties. Transfusion. 2010;50(11):2393–401. 10.1111/j.1537-2995.2010.02730.x . 20561300

[35] Holovati JL, Wong KA, Webster JM, Acker JP. The effects of cryopreservation on red blood cell microvesiculation, phosphatidylserine externalization, and CD47 expression. Transfusion. 2008;48(8):1658–68. 10.1111/j.1537-2995.2008.01735.x . 18482179

[36] Umlas J, Jacobson M, Kevy SV. Suitable survival and half-life of red cells after frozen storage in excess of 10 years. Transfusion. 1991;31(7):648–9. . 1891794 10.1046/j.1537-2995.1991.31791368344.x

[37] Doctor A, Stamler J.S. Nitric Oxide Transport in Blood: A Third Gas in the Respiratory Cycle. 2011. 10.1002/cphy.c090009 23737185

[38] Meryman HT, Hornblower M. A method for freezing and washing red blood cells using a high glycerol concentration. Transfusion. 1972;12(3):145–56. . 5026166 10.1111/j.1537-2995.1972.tb00001.x

[39] Lusianti RE, Benson JD, Acker JP, Higgins AZ. Rapid removal of glycerol from frozen-thawed red blood cells. Biotechnol Prog. 2013;29(3):609–20. 10.1002/btpr.1710 . 23436802

[40] Usry RT, Moore G.L., Manalo F.W. Morphology of Stored, Rejuvenated Human Erythrocytes. Vox sanguinis. 1975;28(3):176–83. 1119131 10.1111/j.1423-0410.1975.tb02756.x

[41] Zhu H, Zennadi R, Xu BX, Eu JP, Torok JA, Telen MJ, et al Impaired adenosine-5'-triphosphate release from red blood cells promotes their adhesion to endothelial cells: a mechanism of hypoxemia after transfusion. Critical care medicine. 2011;39(11):2478–86. 10.1097/CCM.0b013e318225754f 21765360 PMC3196852

[42] Bennett-Guerrero E, Veldman TH, Doctor A, Telen MJ, Ortel TL, Reid TS, et al Evolution of adverse changes in stored RBCs. Proceedings of the National Academy of Sciences of the United States of America. 2007;104(43):17063–8. 10.1073/pnas.0708160104 . 17940021 PMC2040393

[43] Dosier LBM, Premkumar VJ, Zhu H, Akosman I, Wempe MF, McMahon TJ. Antagonists of the system L neutral amino acid transporter (LAT) promote endothelial adhesivity of human red blood cells. Thrombosis and haemostasis. 2017;117(7):1402–11. 10.1160/TH16-05-0373 28382373 PMC5755361

[44] Zhu H, Riccio D, Kirby B, McMahon TJ. Effect of Red Blood Cell ATP Augmentation on Post-Transfusion RBC Extravasation and Changes in Oxygenation in LPS-Exposed Mice. American journal of respiratory cell and molecular biology. 2013;187:A3714.

[45] Pinder AG, Rogers SC, Morris K, James PE. Haemoglobin saturation controls the red blood cell mediated hypoxic vasorelaxation. Advances in experimental medicine and biology. 2009;645:13–20. Epub 2009/02/21. 10.1007/978-0-387-85998-9_3 . 19227444

[46] James PE, Lang D, Tufnell-Barret T, Milsom AB, Frenneaux MP. Vasorelaxation by red blood cells and impairment in diabetes: reduced nitric oxide and oxygen delivery by glycated hemoglobin. Circulation research. 2004;94(7):976–83. 10.1161/01.RES.0000122044.21787.01 . 14963010

[47] Gow A, Doctor A, Mannick J, Gaston B. S-Nitrosothiol measurements in biological systems. Journal of chromatography. 2007;851(1–2):140–51. 10.1016/j.jchromb.2007.01.052 . 17379583 PMC1949323

[48] McMahon TJ, Moon RE, Luschinger BP, Carraway MS, Stone AE, Stolp BW, et al Nitric oxide in the human respiratory cycle. Nature medicine. 2002;8(7):711–7. 10.1038/nm718 . 12042776

[49] Jia L, Bonaventura C, Bonaventura J, Stamler JS. S-nitrosohaemoglobin: a dynamic activity of blood involved in vascular control. Nature. 1996;380(6571):221–6. 10.1038/380221a0 . 8637569

[50] D’Alessandro A, Nemkov T., Yoshida T., Bordbar A., Palsson B.O., and Hansen K.C. Citrate metabolism in red blood cells stored in additive solution-3. Transfusion. 2017;57(2):325–36. Epub 2016 Nov 4. 10.1111/trf.13892 27813142

[51] Reisz JA, Wither M.J., Dzieciatkowska M., Nemkov T., Issaian A., Yoshida T., et al Oxidative modifications of glyceraldehyde 3-phosphate dehydrogenase regulate metabolic reprogramming of stored red blood cells. Blood. 2016;128(12):e32–e42. 10.1182/blood-2016-05-714816 27405778

[52] Corbisier P, Houbion A, Remacle J. A new technique for highly sensitive detection of superoxide dismutase activity by chemiluminescence. Analytical biochemistry. 1987;164(1):240–7. . 2823632 10.1016/0003-2697(87)90392-7

[53] Souza HP, Liu X, Samouilov A, Kuppusamy P, Laurindo FR, Zweier JL. Quantitation of superoxide generation and substrate utilization by vascular NAD(P)H oxidase. American journal of physiology Heart and circulatory physiology. 2002;282(2):H466–74. 10.1152/ajpheart.00482.2001 . 11788393

[54] Clasquin MF, Melamud E., and Rabinowitz J.D. LC-MS data processing with MAVEN: a metabolomic analysis and visualization engine. Current Protocols in Bioinformatics. 2012;37(1):14.1.1-.1.23. 10.1002/0471250953.bi1411s37 PMC4055029 22389014

[55] Chong J, Othman S., Li C., Caraus I., Li S., Bourque G., et al MetaboAnalyst 4.0: towards more transparent and integrative metabolomics analysis. Nucleic acids research. 2018;46(W1):W486–W94. 10.1093/nar/gky310 29762782 PMC6030889

[56] Palmer GM, Fontanella AN, Shan S, Hanna G, Zhang G, Fraser CL, et al In vivo optical molecular imaging and analysis in mice using dorsal window chamber models applied to hypoxia, vasculature and fluorescent reporters. Nature protocols. 2011;6(9):1355–66. 10.1038/nprot.2011.349 21886101 PMC3500601

[57] Schneider CA, Rasband WS, Eliceiri KW. NIH Image to ImageJ: 25 years of image analysis. Nat Methods. 2012;9(7):671–5. 22930834 10.1038/nmeth.2089 PMC5554542

[58] Alshaiban A, Muralidharan-Chari V., Nepo A., and Mousa S.A. Modulation of Sickle Red Blood Cell Adhesion and its Associated Changes in Biomarkers by Sulfated Nonanticoagulant Heparin Derivative Clinical and Applied Thrombosis/Hemostasis. 2015;22(3):230–8. 10.1177/1076029614565880 25601897

[59] Zennadi R, Moeller B.J., Whalen E.J., Batchvarova M., Xu K., Shan S., et al Epinephrine-induced activation of LW-mediated sickle cell adhesion and vaso-occlusion in vivo. Blood. 2007;110(7):2708–17. 10.1182/blood-2006-11-056101 17609430 PMC1988948

[60] Waymouth C. Osmolality of mammalian blood and of media for culture of mammalian cells. In Vitro. 1970;6:109–27. 4943053 10.1007/BF02616113

[61] Silverstein E, Sokoloff L., Mickelsen O., and Jay G.E. Primary polydipsia and hydronephrosis in an inbred strain of mice. American Journal of Pathology. 1961;38(2):143–59. 19970997 PMC1945000

[62] Hall NH, Isaza R., Hall J.S., Wiedner E., Conrad B.L., and Wamsley H.L. Serum osmolality and effects of water deprevation in captive Asian elephants (Elephas maximus). Journal of Veterinary Diagnostic Investigation. 2012;24(4):688–95. 10.1177/1040638712445770 22643341 PMC3886624

[63] Wolk E. Erythrocytes, haemoglobin and haematocrit in the postnatal development of the root vole. Acta Theriol. 1970;15:283–93.

[64] Wolk E. Variations in the hematological parameters of shrews. Acta Theriol. 1974;19:315–46.

[65] Wolk E. The hematology of the free-ranging European bison. Acta Theriol. 1983;28:73–82.

[66] Wolk E. Hematology of a hibernating rodent—the northern birch mouse Acta Theriol. 1985;30:337–48.

[67] Kostelecka-Myrcha A. Hemoglobin, erythrocytes and hematocrit in the blood of some microtidae under laboratory conditions. Bull Acad Pol Sci Biol. 1966;14(5):343–9. 5960146

[68] Turgeon ML. Clinical Hematology: Theory and Procedure. Philadelphia, PA: Lippincott Williams & Wilkinson; 2004.

