# Supplementary material for: Red blood cell phenotype fidelity following glycerol cryopreservation optimized for research purposes
Source: PLoS One. 2018 Dec 21;13(12):e0209201. doi: 10.1371/journal.pone.0209201 (PMC6303082; doi:10.1371/journal.pone.0209201)
Supplement: S1 Extended Methods — (DOC) [file pone.0209201.s001.doc]

**S1 EXTENDED METHODS**

**METHODS**

**Standard glycerolization**

We employed a scaled-down step-wise dilution procedure, as previously reported[1], based on a method originally described by Meryman and Hornblower[2, 3]. After centrifugation, plasma and buffy coat were removed; packed RBCs (4ml) were drawn from the bottom of the cell pellet **(to ensure sampling furthest from the buffy coat)** and glycerolized in two steps at room temperature, using an aqueous cryopreservation solution comprising: glycerol (57.1 grams) with KCl (5mM), MgCl_2_ (5mM), NaC_3_H_5_O_3_ (140mM) and Na_2_HPO_4_ (5mM, final pH 6.8). In the first step, this cryopreservation solution (1.5ml) was added dropwise (3min); the mixture was then allowed to equilibrate undisturbed (5 min). In the second step, additional glycerol solution (5 mL) was added dropwise (3min), yielding a final glycerol concentration ~ 40% (w/v). Total processing time was ~ 25min. Glycerolized samples were then placed into a Coolcell freezing container (Biocision, Larkspur, CA), ensuring rate-controlled freezing (-1°C/min when placed into a -80°C freezer).

**Adapted glycerolization**

**We adapted the above cryopreservation process to simplify and** shorten sample processin**g and to allow processing,** collection and storage of smaller RBC sample volumes**. Initial collection and centrifugation were unchanged from the standard glycerolization protocol. At room temperature, RBCs (300μl) were aliquoted from the bottom of the RBC pellet into cryopreservation tubes containing 300μl of PBS (NaCl 146mM, KCl 2.68mM,** Na_2_HPO_4_ 10.1mM, KH_2_PO_4_ 1.76mM, DTPA 500μM and glucose 6mM, pH 7.4) and 300μl PBS-glycerol (same as the PBS solution, but containing glycerol at 60% v/v). Immediately upon addition of RBCs to PBS, samples were mixed gently by repeated inversion, after which bolus PBS-glycerol (900μl) was added to each tube and the samples were inverted once again (glycerol final was 40% v/v). Glycerolized samples were then placed into a Coolcell freezing container (Biocision, Larkspur, CA), ensuring rate-controlled freezing (-1°C/min when placed into a -80°C freezer). Matched fresh RBC samples were processed (washed 3X in PBS, unless stated otherwise) for immediate analysis.

**Standard deglycerolization**

We employed a consolidated serial dilution procedure[1], based upon the original method described by Meryman and Hornblower[2]. Deglycerolizing solutions consisted of 12% NaCI, 1.6% NaCI (unbuffered), followed by an isotonic wash (0.8% NaCI, 0.2% glucose (11mM), pH 7.4). First, 12% NaCl (50μl, over 5min [10μl/min]) was added to thawed glycerolized blood (~ 400μl, 75% Hct). The resulting mixture was equilibrated undisturbed (3min), then further diluted by addition of 1.6% NaCl (475μl, over 5min [95μl/min]). RBCs were then pelleted, (1,500*g*, 5min) and re-suspended in the isotonic wash (500μl, over 5min [100μl/min]), and re-pelleted (1,500*g*, 5min) and repeated (3X, total). The entire deglycerolization process was carried out at RT and required approximately 45mins for completion.

Adapted deglycerolization

We optimized the above method to minimize RBC loss to lysis by extending the dilution sequence (reducing RBC suspension osmolality less rapidly). First, 12% NaCl (50 μL, over 5min) was added to thawed, glycerolized blood (~400μl, 75% Hct). The resulting mixture was allowed to equilibrate undisturbed (3min) and then was further diluted by addition of 1.6% NaCl (475μL, over 5min), after which 1.6% NaCl (1ml, over 5min) was added, allowed to briefly equilibrate, then further diluted with additional 1.6% NaCl (2ml, over 5min). At this stage, samples were transferred to 15ml conical tubes, to which we added the isotonic wash (2.5ml, 0.8% NaCl, 0.2% (11mM) glucose, pH 7.4, over 5min). After brief equilibration, additional isotonic wash was added (8ml, over 8min). The RBC suspension was then pelleted (1,500*g*, 5min) and resuspended (10ml bolus addition of a 3:1 mix of 1.6% NaCl and the isotonic wash), re-pelleted and then resuspended/re-pelleted in pure isotonic wash (10ml). The entire deglycerolization process was carried out at RT and required ~ 1hour for completion.

RBC lysis

An alternative approach to calculate the %age of RBC lysis is to divide the (post-centrifugation) supernatant Hb concentration (numerator) by the RBC suspension Hb concentration (denominator) and multiply by 100, ignoring the volumes. Ignoring the volumes is reasonable because: a) they are similar (the suspension RBC hematocrit is <20%), and b) accurate measurement of pellet volume is difficult, because either a thin layer of supernatant typically remains over the pellet after supernatant aspiration, or else the pellet is touched. Others have noted that in low-hematocrit systems like ours, correction for the hematocrit has little effect on the % lysis results[4]. We found close agreement (~15%) between the two methods when applied to a set of thawed samples that varied in lysis degree.

**Intracellular RBC glycerol**

Intracellular RBC glycerol was determined using a commercially available Glycerol assay kit (Sigma MAK117). Briefly, packed RBCs (40μl) were added to ddH_2_O (660μl) and 5% metaphosphoric acid (MPA, 300μl), vortexed vigorously, then centrifuged (25,000*g*, 10min, 4°C) to pellet protein; clear supernatant (10μl) was added to a 96 well plate with the master reaction mix (assay buffer, enzyme mix, ATP, dye reagent) (100μl). Samples were incubated (20min, RT), protected from light, and then measured spectrophotometrically (A_570_; Biotek μQuant, Winooski, VT, USA).

Scanning Electron Microscopy

For sample analysis via Scanning Electron Microscopy (SEM), 12mm diameter poly-L-lysine coated coverslips were placed into a 12-well plate with each well filled with 1ml of 0.85% sodium chloride so as to submerge each coverslip. Diluted RBC samples (50µl) were gently transferred via pipette onto the top of each coverslip and left to incubate for 5min. A 1ml solution of 4% paraformaldehyde and 0.4% glutaraldehyde in 0.2M cacodylate buffer was then added to each well and the samples were allowed to fix for 1hour. Post fixation, each sample coated coverslip was rinsed in ultrapure water 3 times for 10min each followed by a secondary fixation in an aqueous solution of 1% OsO_4_ overnight at 4˚C. Post-secondary fixation, the sample coverslips were then rinsed again 3 times in ultrapure water for 10min each and dehydrated in a graded ethanol series (30%, 40%, 50%, 60%, 70%, 80%, 90%, 100%) for 10min at each step. Once dehydration was complete, the sample coverslips were loaded into a critical point drier (Leica EM CPD 300, Vienna, Austria), which was set to perform 16 CO_2_ exchanges at the slowest speed. Once dried, the sample coverslips were then mounted on aluminum stubs with carbon adhesive tabs and sputter coated with 10nm of iridium (Leica ACE 600, Vienna, Austria). After iridium coating, the sample coverslips were loaded into a FE-SEM (Zeiss Merlin, Oberkochen, Germany) and imaged at 3KeV with a probe current of 300pA using the Everhart Thornley secondary electron detector.

RBC adhesion (*ex vivo*)

RBC adhesivity to endothelium was determined as previously described[5-8]: RBCs labeled with the fluorescent dye PKH26 were perfused in a custom-made variable-height plexiglass flow chamber over glass slides supporting human umbilical vein endothelial cells (HUVECs) grown to confluence[9]. The fluid height was measured at seven different points along the chamber. The RBC sample was introduced to the chamber at a rate of 1.5ml min^-1^, then allowed to dwell for 5–10 min. The number of cells at each location (height) was recorded. After the static phase, 5–10 min of fluidic flow was conducted with PBS at flow rates calculated to produce the desired shear stress range of ~1–10dynes/ cm^2^. Following exposure to flow, the number of adherent cells at each location was counted. Shear stress and percent adhesion were calculated at each height.

Oxygen affinity

Oxygen-hemoglobin dissociation curves were determined *in vitro* (Hemox-Analyzer, TSC Scientific Corporation, New Hope, PA, USA). Briefly, the Hemox uses a Clark type O_2_ electrode in combination with dual-wavelength spectrophotometry (560 and 576nm) to simultaneously measure pO_2_ (Torr) and hemoglobin O_2_ saturation (HbSO_2_%). Fresh washed (or deglycerolized) pRBCs (25μL) were diluted in BIS TRIS (5ml, BIS TRIS 50mM, NaCl 100mM; buffered to either pH 7.2, 7.4, or 7.6) [10], with addition of bovine serum albumin BSA (20μL), and an antifoaming agent (10μL), both supplied by the manufacturer. Samples were placed within the machine cuvette and equilibrated to 37°C, while bubbled with air from an attached cylinder (to fully oxygenate). Samples were subsequently deoxygenated by exposure to N_2_, while monitoring both HbSO_2_ and pO_2_. The Hb p50 value (pO_2_ at which the HbSO_2_ is 50%) was extrapolated from the plotted relationship of the above two variables (oxy-hemoglobin desaturation curve); in addition, the Hill co-efficient was also calculated from these data (TCS Hemox Data Acquisition System, TCS Scientific Corp, New Hope, PA, USA), providing an index of cooperativity.

RBC vasoactivity (hypoxic vasodilation, HVD)

Male New Zealand white rabbits (1.8 to 2Kg) were euthanized by intravenous injection of sodium pentobarbital. The aorta was harvested and endothelium intact rings were prepared for isometric tension recordings by mounting on a Radnotti vascular ring array (Harvard Apparatus, Holliston, MA, USA): 2grams resting tension, 37°C, Krebs, as described[11]. Rings were pre-conditioned at 95% O_2_, 5% CO_2_, with phenylephrine (PE) 10^-6^ mol/L and acetylcholine (ACh) 10^-5^ mol/L; then at low oxygen tension (95% N_2_, 5% CO_2_; ~1% O_2_), PE (5x10^-6^ mol/L) was used to increase baseline tension, before 40μL of pelleted RBCs were injected into each bath. Endothelium-intact rings were identified as those demonstrating an ACh relaxation response during preconditioning >60% of the maximal induced tension (by PE). Any rings not producing this amount of relaxation were excluded from analysis. The resulting change in isometric tension was recorded by transducers linked to a Powerlab 8Sp/octal bridge (AD Instruments, Colorado Springs, CO, USA) connected to a PC running LabChart 7 (AD Instruments, Colorado Springs, CO, USA). For each experiment, eight aortic ring preparations were run in parallel, n=1 represents data averaged from all the endothelium intact rings (possible 8 total) that were treated identically; the % relaxation was calculated as the RBC-induced decrease in tension as a percentage of the preceding baseline plateau tension (both during the above hypoxic bath conditions)[12].

RBC nitric oxide content

Fresh RBC samples, frozen RBC samples (without cryopreservation) and cryopreserved RBC Hb samples (deglycerolized or not) were assayed for total, Fe-bound and thiol-bound NO (SNO) content by photolysis/chemiluminescence, as described previously[13]. Briefly, samples were injected into a custom photolysis system (Technosoft, Morrisville, NC, USA) to photolytically liberate bound NO that was carried by a helium gas stream to a high-resolution chemiluminescence NO analyzer (TEA 810, Ellutia, Charleston, SC, USA). Aqueous phase and higher oxides of nitrogen were removed in a series of cold traps interposed between the photolysis and analyzer units. No sample treatment was used, other than desalting on G25 spin columns and paired analysis of samples following addition of HgCl_2_ (6 fold molar excess over [Hb thiol]; tetramer basis) or vehicle[14, 15].

Metabolomics

Metabolomics analyses and tracing experiments with [1,2,3-^13^C_3_]glucose were performed as previously reported[16, 17]. Metabolite standard reagents were purchased from Sigma Aldrich (St. Louis, MO) or Cambridge Isotope Laboratories, Inc. (Tewksbury, MA).

Either washed fresh red blood cells (RBCs) or glycerolized, frozen, thawed and deglycerolized RBCs (denoted deglycerolized; DG) were incubated with [1,2,3-^13^C_3_]glucose (no. CLM-4673-PK) in the presence of (i) PBS (control), (ii) DMSO (vehicle control), or (iii) the oxidant SOTS1 (superoxide producer), dosed to generate superoxide levels in the physiologically relevant range produced by activated vascular NADPH oxidase[18, 19]. Sequential sampling of cells and supernatants was performed at 0, 1, 3 and 6 hours, at which time cells and supernatants were separated (2,000*g*, 10 min, 4°C). Catabolism of [1,2,3-^13^C_3_]glucose generates distinct isotopologues of lactate, either M+2 and M+3, depending on whether glucose oxidation preferentially occurs through the pentose phosphate pathway (PPP) or the Embden-Meyerhof-Parna (EMP) glycolytic pathway.

Samples were prepared for UHPLC-MS metabolomics in the following manner. RBCs (25µl) and supernatants (25µl) were extracted in ice-cold lysis/extraction buffer (methanol:acetonitrile:water 5:3:2) at 1:10 and 1:25 dilutions, respectively. Samples were then agitated (30min, 4°C) followed by centrifugation (10,000*g*, 15min, 4°C). Protein pellets were discarded, while supernatants were stored at -80°C prior to metabolomic analyses. RBC and supernatant extracts (10µl) were injected into an UHPLC system (Vanquish, Thermo, San Jose, CA, USA) and run on a Kinetex XB-C18 column (150 x 2.1mm, 1.7µm – Phenomenex, Torrance, CA, USA), as previously reported [16]. Technical mixes were generated by pooling aliquots of cell and supernatant extracts (separately), and were run every 15 analytical runs to control for technical variability, determined as coefficients of variation (CV). CV were determined by calculating the ratios of standard deviations divided by mean measurements for compounds of interest (e.g. all isotopologues of lactate) across all technical mix runs. Technical reproducibility was also tested by determining Coefficients of Variation for the quantitation of spiked xenometabolite 5-fluorouracil (1µM final concentration – no. F6627-1G – Sigma Aldrich, St. Louis, MO, USA) across all tested samples and in technical mixes. All metabolites reported in this study had CV<10% in all tested mixes (cells and supernatants).

The Q Exactive mass spectrometer (Thermo, Bremen, Germany) was operated in Full MS mode (2µscans) at 70,000 resolution in the 60-900m/z range, 4kV spray voltage, 15 sheath gas and 5 auxiliary gas in positive and negative ion modes (separate runs). Metabolite assignments and isotopologue distributions were determined using Maven (Princeton, NJ, USA)[20] and assignments confirmed against 700 standards (IROATech, Sigma Aldrich, St. Louis, MO, USA).

In vivo analysis of RBC adhesion, RBC velocity, and Hb saturation

Nude mice (*Foxn1^nu^;* Jackson Labs, strain 002019) were anesthetized (isoflurane) and fitted with a dorsal window chamber, as we described[21]. After recovery (1-3 days), mice were anesthetized (isoflurane), placed upon a temperature-controlled platform and inspected to verify intact microcirculation. PKH-26 labeled RBCs (10μL/g body weight) were infused over 10sec and label movement was tracked by intravital microscopy using a 5X objective (Zeiss Axioobserver Z1, Zeiss Zen software). Videos (transmission and red fluorescent channel) and still images of the microvasculature were captured for each subject, and analyzed offline for RBC adhesion, RBC velocity, and Hb saturation (ImageJ, available free, NIH)(MatLab R2017b, Mathworks)[22] and custom analysis software.

Adhesion was measured by time-averaging an image sequence of ~60sec, yielding a composite image in which adherent cells appeared as distinct fluorescent entities, while any moving cells blur out and appear as diffuse grey background signal. Individual adherent RBCs were then hand counted. A random representative still frame image was then selected from each corresponding video image. The total number of cells in one frame was also counted using automated ImageJ particle-counting software. Adhesion values for each subject were calculated and presented as the percent adherent cells relative to the total cells present in a single video frame.

To measure RBC velocity, five venules of various sizes were randomly selected from each subject’s video. Individual RBCs were selected from these venules, then tracked through 5-10 frames of movement. Because the velocity of individual RBCs through each particular venule is not uniform, two cells were selected from a particular segment of each venule and their velocities through a shared segment of that venule were averaged. The diameter of each venule was measured perpendicular to the mid-point of the path of travel of the two cells. Each two-cell velocity average was then normalized to the inverse square of the radius of the venule segment through which the cells passed. This provided five normalized RBC velocities per subject which were then averaged to get a single (n=1) average radius^2^-normalized RBC velocity per subject. The paths of the RBCs were recorded on separate .roi files for utilization in Hb saturation analysis.

To measure Hb saturation still images of each subject were taken at a range of wavelengths and processed using Matlab to generate an Hb saturation % heat map of a region of venules within the dorsal window[21]. For each individual subject these images were taken at the same time and in the same region as the videos used to perform the RBC velocity and adhesion analysis. The individual .roi files containing the paths of the RBCs were then overlaid over these maps. The segments of each venule over which the RBCs traveled during their velocity analysis were then isolated. The Hb saturation percentages were then averaged within each segment. The five segment Hb saturation values for each subject were then averaged to give a final averaged Hb saturation percentage value for each subject.

**REFERENCES**

1. Lusianti RE, Benson JD, Acker JP, Higgins AZ. Rapid removal of glycerol from frozen-thawed red blood cells. Biotechnol Prog. 2013;29(3):609-20. doi: 10.1002/btpr.1710. PubMed PMID: 23436802.

2. Meryman HT, Hornblower M. A simplified procedure for deglycerolizing red blood cells frozen in a high glycerol concentration. Transfusion. 1977;17(5):438-42. PubMed PMID: 910260.

3. Meryman HT, Hornblower M. A method for freezing and washing red blood cells using a high glycerol concentration. Transfusion. 1972;12(3):145-56. PubMed PMID: 5026166.

4. Sowemimo-Coker. Red blood cell hemolysis. Transfusion Medicine Reviews. 2002;16(1):46-60.

5. Zhu H, Zennadi R, Xu BX, Eu JP, Torok JA, Telen MJ, et al. Impaired adenosine-5'-triphosphate release from red blood cells promotes their adhesion to endothelial cells: a mechanism of hypoxemia after transfusion. Critical care medicine. 2011;39(11):2478-86. doi: 10.1097/CCM.0b013e318225754f. PubMed PMID: 21765360; PubMed Central PMCID: PMCPMC3196852.

6. Bennett-Guerrero E, Veldman TH, Doctor A, Telen MJ, Ortel TL, Reid TS, et al. Evolution of adverse changes in stored RBCs. Proceedings of the National Academy of Sciences of the United States of America. 2007;104(43):17063-8. PubMed PMID: 17940021.

7. Dosier LBM, Premkumar VJ, Zhu H, Akosman I, Wempe MF, McMahon TJ. Antagonists of the system L neutral amino acid transporter (LAT) promote endothelial adhesivity of human red blood cells. Thrombosis and haemostasis. 2017;117(7):1402-11. doi: 10.1160/TH16-05-0373. PubMed PMID: 28382373; PubMed Central PMCID: PMCPMC5755361.

8. Zhu H, Riccio D, Kirby B, McMahon TJ. Effect of Red Blood Cell ATP Augmentation on Post-Transfusion RBC Extravasation and Changes in Oxygenation in LPS-Exposed Mice. American journal of respiratory cell and molecular biology. 2013;187:A3714.

9. Wada Y, Otu H, Wu S, Abid MR, Okada H, Libermann T, et al. Preconditioning of primary human endothelial cells with inflammatory mediators alters the "set point" of the cell. FASEB J. 2005;19(13):1914-6. doi: 10.1096/fj.05-4037fje. PubMed PMID: 16172186; PubMed Central PMCID: PMCPMC5378497.

10. Benesch RE, Benesch R, Yu CI. The oxygenation of hemoglobin in the presence of 2,3-diphosphoglycerate. Effect of temperature, pH, ionic strength, and hemoglobin concentration. Biochemistry. 1969;8(6):2567-71. PubMed PMID: 5799137.

11. Pinder AG, Rogers SC, Morris K, James PE. Haemoglobin saturation controls the red blood cell mediated hypoxic vasorelaxation. Advances in experimental medicine and biology. 2009;645:13-20. Epub 2009/02/21. doi: 10.1007/978-0-387-85998-9_3. PubMed PMID: 19227444.

12. James PE, Lang D, Tufnell-Barret T, Milsom AB, Frenneaux MP. Vasorelaxation by red blood cells and impairment in diabetes: reduced nitric oxide and oxygen delivery by glycated hemoglobin. Circulation research. 2004;94(7):976-83. PubMed PMID: 14963010.

13. Gow A, Doctor A, Mannick J, Gaston B. S-Nitrosothiol measurements in biological systems. Journal of chromatography. 2007;851(1-2):140-51. PubMed PMID: 17379583.

14. McMahon TJ, Moon RE, Luschinger BP, Carraway MS, Stone AE, Stolp BW, et al. Nitric oxide in the human respiratory cycle. Nature medicine. 2002;8(7):711-7. PubMed PMID: 12042776.

15. Jia L, Bonaventura C, Bonaventura J, Stamler JS. S-nitrosohaemoglobin: a dynamic activity of blood involved in vascular control. Nature. 1996;380(6571):221-6. PubMed PMID: 8637569.

16. D’Alessandro A, Nemkov, T., Yoshida, T., Bordbar, A., Palsson, B.O., and Hansen, K.C. Citrate metabolism in red blood cells stored in additive solution-3. Transfusion. 2017;57(2):325-36. Epub 2016 Nov 4.

17. Reisz JA, Wither, M.J., Dzieciatkowska, M., Nemkov, T., Issaian, A., Yoshida, T., Dunham, A.J., Hill, R.C., Hansen, K.C., and D’Alessandro, A. Oxidative modifications of glyceraldehyde 3-phosphate dehydrogenase regulate metabolic reprogramming of stored red blood cells. Blood. 2016;128(12):e32-e42.

18. Corbisier P, Houbion A, Remacle J. A new technique for highly sensitive detection of superoxide dismutase activity by chemiluminescence. Analytical biochemistry. 1987;164(1):240-7. PubMed PMID: 2823632.

19. Souza HP, Liu X, Samouilov A, Kuppusamy P, Laurindo FR, Zweier JL. Quantitation of superoxide generation and substrate utilization by vascular NAD(P)H oxidase. American journal of physiology Heart and circulatory physiology. 2002;282(2):H466-74. PubMed PMID: 11788393.

20. Clasquin MF, Melamud, E., and Rabinowitz, J.D. LC-MS data processing with MAVEN: a metabolomic analysis and visualization engine. Current Protocols in Bioinformatics. 2012;37(1):14.1.1-.1.23.

21. Palmer GM, Fontanella AN, Shan S, Hanna G, Zhang G, Fraser CL, et al. In vivo optical molecular imaging and analysis in mice using dorsal window chamber models applied to hypoxia, vasculature and fluorescent reporters. Nature protocols. 2011;6(9):1355-66. doi: 10.1038/nprot.2011.349. PubMed PMID: 21886101; PubMed Central PMCID: PMCPMC3500601.

22. Schneider CA, Rasband WS, Eliceiri KW. NIH Image to ImageJ: 25 years of image analysis. Nat Methods. 2012;9(7):671-5. PubMed PMID: 22930834; PubMed Central PMCID: PMCPMC5554542.
