# Supplementary material for: Red blood cell phenotype fidelity following glycerol cryopreservation optimized for research purposes
Source: PLoS One. 2018 Dec 21;13(12):e0209201. doi: 10.1371/journal.pone.0209201 (PMC6303082; doi:10.1371/journal.pone.0209201)

**S2 FIGURE 1**

**Figure 1.** **RBC morphological imaging (scanning electron microscope; SEM) and measurement (complete blood count; CBC) for fresh RBCs and for RBCs following glycerol addition, thawing after freezing, and full deglycerolization.** Illustration of the characteristic biconcave disc morphology of **(A column 1)** fresh RBCs. **(A Column 2)** Following glycerol addition, the majority of RBCs became swollen and spherocytic. **(A Column 3)** After thawing, RBCs remained encapsulated in a glycerol matrix. **(A Column 4)** Following deglycerolization, RBCs returned to their original morphology. **(B)** Mean corpuscular volume (MCV) and **(E)** red cell distribution width (RDW) significantly increased following addition of glycerol to fresh RBCs. **(C)** No change was observed in mean corpuscular hemoglobin (MCH), while **(D)** mean corpuscular hemoglobin concentration (MCHC) declined (n = 5 individual blood donors). Following full deglycerolization, all parameters return to levels observed in fresh samples.

Figure 1.


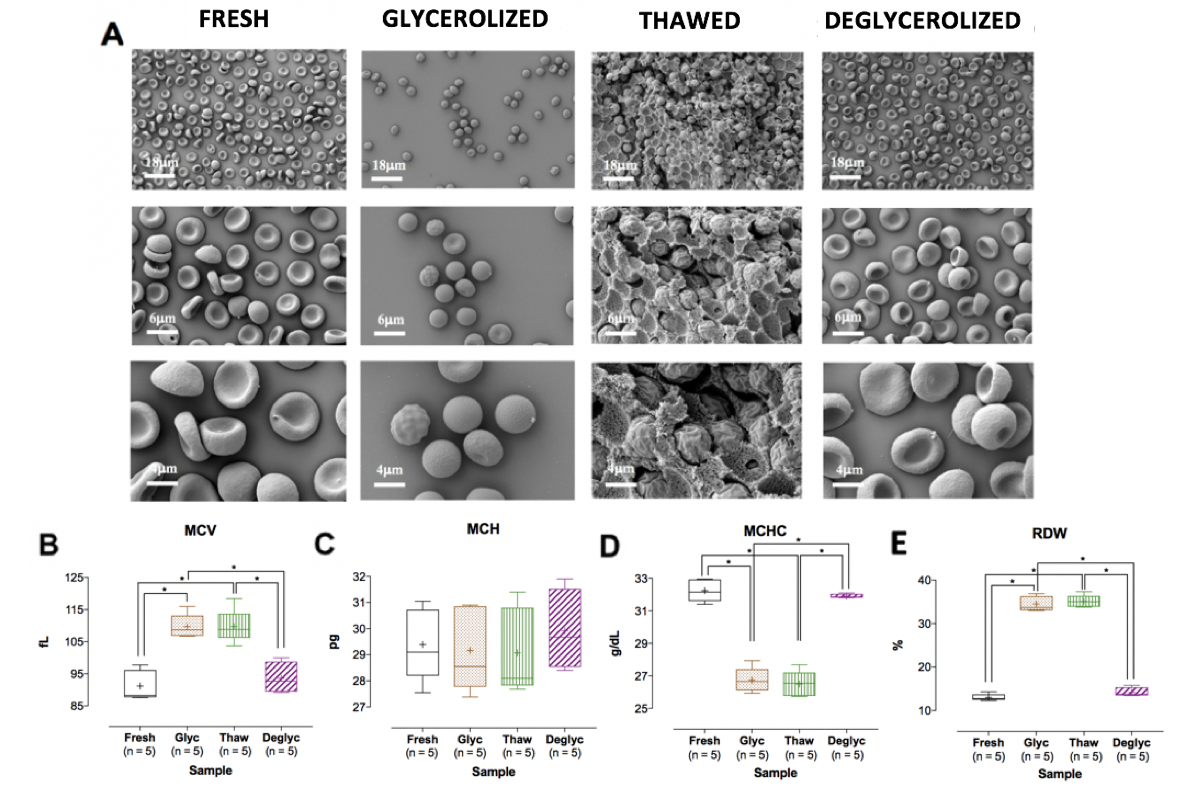

Supplement: S1 Fig — (DOC) [file pone.0209201.s002.doc]
