# Supplementary material for: Red blood cell phenotype fidelity following glycerol cryopreservation optimized for research purposes
Source: PLoS One. 2018 Dec 21;13(12):e0209201. doi: 10.1371/journal.pone.0209201 (PMC6303082; doi:10.1371/journal.pone.0209201)
Supplement: S2 Fig — (DOC) [file pone.0209201.s003.doc]

**S3 FIGURE 2**

**Figure 2. Heat map with named metabolites.** Visual indication of a time course and SOTS dependency of the metabolic phenotypes observed in matched fresh and deglycerolized RBCs.


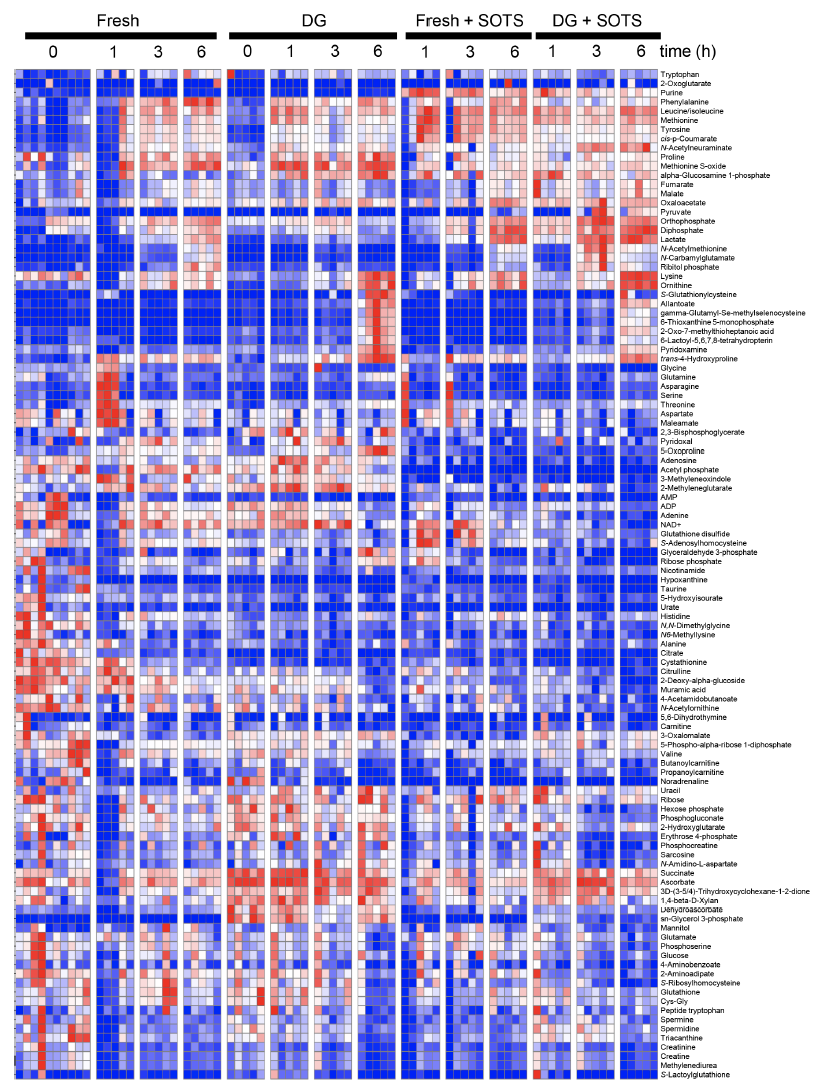
Figure 2.
